# Dual anaerobic reactor model to study biofilm and microbiologically influenced corrosion interactions on carbon steel

**DOI:** 10.1038/s41529-024-00542-x

**Published:** 2024-12-06

**Authors:** Liam Jones, Maria Salta, Torben Lund Skovhus, Kathryn Thomas, Timothy Illson, Julian Wharton, Jeremy Webb

**Affiliations:** 1 School of Biological Sciences, University of Southampton, Southampton, UK; 2 Endures, MIC and Biofilm Department, Bevesierweg 1, DC002, 1781 AT Den Helder, The Netherlands; 3 School of the Environment and Life Sciences, University of Portsmouth, Portsmouth, UK; 4 Research Centre for Built Environment, Climate and Water Technology, VIA University College, Horsens, Denmark; 5 DNV, Holywell Park, Ashby Road, Loughborough, UK; 6 School of Engineering, University of Southampton, Southampton, UK; 7 National Biofilms Innovation Centre, Southampton, UK

**Keywords:** Microbiology, Engineering, Materials science

## Abstract

Continual challenges due to microbial corrosion are faced by the maritime, offshore renewable and energy sectors. Understanding the biofilm and microbiologically influenced corrosion interaction is hindered by the lack of robust and reproducible physical models that reflect operating environments. A novel dual anaerobic biofilm reactor, using a complex microbial consortium sampled from marine littoral sediment, allowed the electrochemical performance of UNS G10180 carbon steel to be studied simultaneously in anaerobic abiotic and biotic artificial seawater. Critically, DNA extraction and 16S rRNA amplicon sequencing demonstrated the principal biofilm activity was due to electroactive bacteria, specifically sulfate-reducing and iron-reducing bacteria.

## Introduction

Microbiologically influenced corrosion (MIC) is a complex process that causes structural and operational degradation, especially in the energy and maritime sectors. Understanding the precise MIC mechanism is not straightforward as there can be numerous competing abiotic and biotic corrosion mechanisms taking place at any given time. As such, understanding and predicting MIC is a particular challenge since there is a lack of robust and reproducible physical biofilm model systems that reflect real-world and operating environments. With the recent operating history of the offshore wind farm industry, the scale problems and associated costs are yet to be fully understood, but it has become apparent the actual environmental corrosivity can be very different from that originally assumed in the design specification. Early monopile design assumptions anticipated low, uniform corrosion rates in sealed compartments that would be completely air- and water-tight. However, operational experience has shown anaerobic (anoxic) microbial conditions can quickly develop negating the original installation protection strategies leading to structural deterioration^1^.

Detailed knowledge of how microorganisms behave within mixed biofilm communities is limited^2–4^. Additionally, the extent to which abiotic or biotic conditions lead to corrosion is also complex. Abiotic corrosion is linked to physical and non-biological factors. Whilst biotic (microbial) corrosion involves the deterioration of a surface because of the metabolic activity of microorganisms, either directly or indirectly, through extracellular electron transport (EET), metabolites or biodegradation. A marine biofilm will typically comprise a mixed-species consortium that forms synergistic relationships, enabling the mixed-species biofilm to be more recalcitrant, compared to single-species biofilms and planktonic microorganisms^5^. A wide diversity of microorganisms, typically growing as biofilms, has been implicated in corrosion. Biofilms are aggregates of microorganisms that are typically surface adherent due to the presence of extracellular polymeric substances (EPS), which consist of proteins, polysaccharides, and nucleic acids^6^. Protection from physical disturbances and antimicrobial agents, along with increased environmental stability, are two important advantages afforded to biofilm communities. Furthermore, these sessile microorganisms within a biofilm have greater access to nutrients and other resources accumulating at surfaces, along with enhanced opportunities for interactions such as horizontal gene transfer and co-metabolism which may greatly influence the community dynamics within a biofilm and the threat of MIC^7,8^.

The broad functional diversity of microorganisms apparent in anaerobic soils, sediments, and subsurface environments plays an important ecological and biogeochemical role in nature and can lead to intrinsically heterogeneous biofilms under simulated conditions. However, it is the presence of electroactive microorganisms, capable of exchanging electrons with their extracellular environment, which play a key role in EET associated MIC^9^. These electroactive microorganisms can be further classified as being either electrogens, capable of donating electrons to natural extracellular electron acceptors, or electrotrophs, capable of accepting electrons from natural extracellular electron donors. However, some electroactive microorganisms can switch between functioning as electrogens or electrotrophs, depending on environmental conditions. The full diversity of electroactive microorganisms is still poorly understood and new electroactive microorganisms are continually being identified^9^. In the context of anaerobic sediments and the surface of corroding metals, the relationship between respiratory and fermentative electrogens can have a significant effect on the electromicrobiome, the surrounding environmental chemistry and any corrosion mechanisms, specifically the extent of MIC^9^.

Electroactive bacteria such as sulfate-reducing bacteria (SRB) and iron-reducing bacteria (IRB) are well characterized for their role in MIC. Sulfate-reducing bacteria obtain energy by oxidizing molecular hydrogen or a range of organic compounds to reduce sulfate (${{\rm{SO}}}_{4}^{2-}$), the terminal electron acceptor, to biogenic hydrogen sulfide (${{\rm{H}}}_{2}{\rm{S}}$)^10^. For SRB, electrons are directly withdrawn from the metal surface through membrane-bound redox proteins for sulfate reduction^11,12^. Since ${{\rm{H}}}_{2}{\rm{S}}$ readily combines with ferrous (${{\rm{Fe}}}^{2+}$) ions from the primary dissolution of iron (${{\rm{Fe}}}^{0}$), the net reaction (including bicarbonate that is readily available in many water systems) is as follows^13^:

1 $$4{\rm{Fe}}+{{\rm{SO}}}_{4}^{2-}+{3{\rm{HCO}}}_{3}^{-}+{5{\rm{H}}}^{+}\to {\rm{FeS}}+{3{\rm{FeCO}}}_{3}+4{{\rm{H}}}_{2}{\rm{O}}$$

This net reaction is commonly referred to as electrical microbiologically influenced corrosion (EMIC) and allows SRB to utilize iron more efficiently as an electron donor by direct uptake of electrons from iron oxidation^12,14^. The formation of corrosion products, such as iron sulfide or iron carbonate, can increase the corrosion rate by generating a localized corrosion cell on the metal surface^11^. In contrast, IRB utilizes ferric ions (${{\rm{Fe}}}^{3+}$) and high valence manganese (${{\rm{Mn}}}^{4+}$) as efficient electron acceptors that are capable of out-competing electron acceptors of lower potential, such as sulfate^15,16^. IRB is thought to accelerate the corrosion of steel by removing the ferric oxide passivating layer through iron respiration. Biomineral dissolution reactions by metal-reducing microorganisms remove oxide layers or force mineral replacement reactions that lead to further dissolution of metals. The rate of dissolution depends on many aspects and parameters, such as the chemical nature of the solvent and solute, temperature, and interfacial surface area^17^.

Existing industrial standards, such as the National Association of Corrosion Engineers (NACE) TM0212-2018^18^, acknowledge that sessile microorganisms are the most important biological component of the microbial ecology of an oilfield or natural gas system. Data based on planktonic microorganisms is of limited value since planktonic microorganisms are not directly representative of sessile microorganisms, which form biofilms and cause MIC^19^. It is, however, recognized that techniques and procedures for studying sessile microorganisms are limited and can produce variable outcomes^19,20^. However, numerous scientific articles over the last 20 years have demonstrated the central role of sessile bacteria and the inconsistency of analysing planktonic bacteria for MIC studies. Various assays and test methods have been used for the investigation, diagnosis, and treatment of MIC. However, chemical, microbiological, and corrosion analyses are often used in isolation. Studies wherein MIC has been holistically investigated are limited. The use of different identification and confirmatory tools, combining multiple lines of evidence (MLOE) to evaluate failures that may involve microorganisms, is critical^21^. However, this multi-disciplinary approach requires expertise in multiple areas. A holistic understanding that accurately evaluates the effects of biocides on biofilms, and not of planktonic microorganisms, is needed. The difficulty comes in how to establish and test biofilms in the lab, what analytical methods to use, what information to collect, and how to interpret the data to determine biocide efficacy^22,23^. To our knowledge, this is the first time all these metrics have been combined in one unified approach^21,24^. The overall aim of this paper is to explore recent advances in biofilm testing and MIC research, for instance, to provide recommendations for future standards being drafted by the Association for Material Protection and Performance (AMPP) for the testing of biocides. It is important to note that this article itself is not intending to serve as a standard. The outcome of the work will feed into a standards committee (SC-22 Biodeterioration) document which aims to establish a standard protocol for ‘Laboratory evaluation of the effect of biocides on biofilms’. The proposed protocol is what will feed into the AMPP standard, as currently, there are no nationally or internationally recognized standards or test methods with which to evaluate control strategies effective against biofilm-mediated corrosion.

This study demonstrates the applicability of a novel dual bioreactor protocol to investigate mixed-species biofilms under conditions that mimic the MIC environment of interest. It is important to note that this is the first experiment in our lab using the proposed experimental setup. Overall, the aim is to develop a reproducible bioreactor-based model for MIC that will be able to: (i) gain new scientific insight and mechanistic MIC understanding that will improve predictive measures, including next-generation sequencing of the MIC-associated microbiome and (ii) make steps toward progressing the first standard model and biofilm-relevant test method for the industry. Moreover, the novelty of this protocol is in using a mixed-species environmental biofilm, which also importantly incorporates a multi-disciplinary approach, using MLOE^24^ to gain a holistic understanding of biofilms and MIC.

## Results

### Visual observations

Over the initial three-day batch phase, the abiotic media had no apparent visual changes, and the coupons maintained the silver-grey appearance of the carbon steel. Once the flow of fresh media was started on day 4, the sterile abiotic reactor ASW media became reddish-brown in colouration with increased turbidity (increasing suspended matter). Generally, the abiotic coupons had a reddish-brown corrosion product over the 28 days. Conversely, after inoculation of the biotic reactor ASW media, a black surface film was present on the steel coupons (day 1), with a low level of turbidity. After the flow of fresh ASW media was started on day 4, the ASW media had visible black particulates settling at the bottom of the reactor, with the bulk media being green in appearance. After two weeks, there was a visible crust at the ASW surface, and the media was black in appearance with high turbidity. Over the following two weeks, up to 28 days, the biotic media was dark green/black in colouration. Upon dismantling of the reactors on day 28, and retrieval of the coupon rods, there was an evident difference in the coupon appearances, see Supplementary Fig. 2. The abiotic surfaces were covered in a thick reddish-brown corrosion product. Whilst the biotic surfaces were only partially covered around the centre with a dark green/black deposit.

### Sulfide analysis

**Fig. 1:**
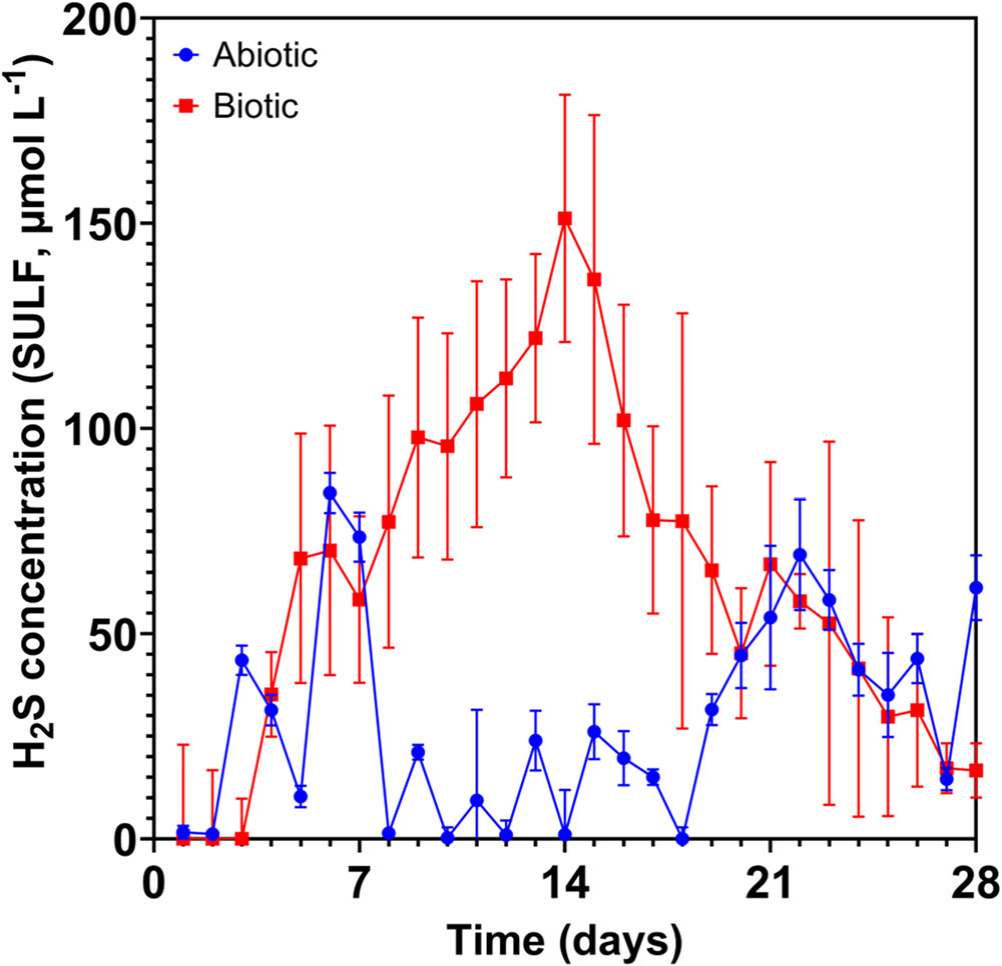
Hydrogen sulfide concentrations over 28 days. Aqueous sulfide measurements (SULF, µmol L^–1^) for the abiotic and biotic conditions over 28 days (nb. measured the anaerobic nutrient-enriched ASW media in situ adjacent to corroding UNS G10180 carbon steel). Hydrogen sulfide concentrations over 28 days.

Figure 1 shows the aqueous sulfide concentrations monitored in the abiotic and the biotic anaerobic nutrient-enriched ASW media over the test duration. For the sterile abiotic condition, there was a generally low sulfide concentration (mean: 29.2 µmol L^–1^). Whereas for the biotic condition, the sulfide concentration steadily increased, resulting in a maximum of about 150 µmol L^–1^ after 2 weeks, before decreasing to similar levels detected for the abiotic media. Dissolved oxygen (DO) (Hanna Instruments) concentrations measured on day 28 were 4.4 ppm in the 10 L media containers, 2.6 ppm in the abiotic and 0.2 ppm in the biotic reactor. The pH was not measured on completion of the experiment.

### Carbon steel surface analysis

**Fig. 2:**
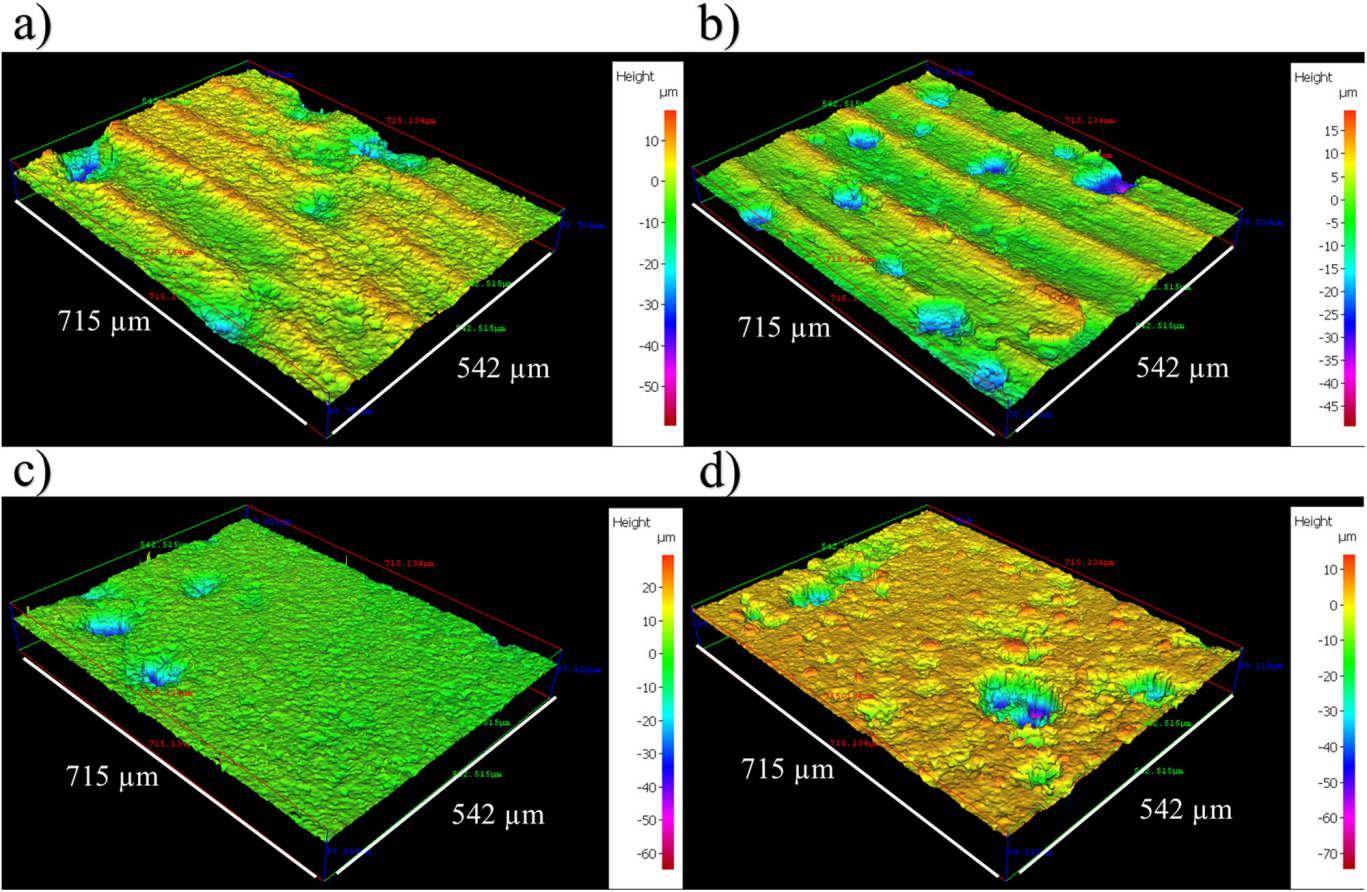
Surface profilometry of carbon steel at day 28. Three-dimensional optical surface profilometry of the cleaned UNS G10180 surfaces at day 28. AR coupons for **a** abiotic and **b** biotic conditions; and P coupons for **c** abiotic and **d** biotic conditions, after exposure to anaerobic nutrient-enriched ASW media for 28 days. Surface Profilometry of carbon steel at day 28.

Supplementary Fig. 3 shows the carbon steel surfaces on day 0. Supplementary Table 3 summarizes the quantitative surface roughness profiles on both day 0 and day 28. Prior to testing, the AR and P coupons had markedly different surface profiles, owing primarily to the clearly evident machine marks on the AR surfaces. The machine marks were still evident on day 28. However, both coupon samples exhibited similar surface roughness profiles on day 28. Figure 2 shows the cleaned carbon steel surfaces after 28 days, with biofilms and corrosion products removed, to reveal the morphology of the surface degradation and to facilitate corrosion assessment. Surface profilometry revealed that both abiotic and biotic anaerobic ASW media led to localized pitting, with the biotic condition more extensively pitted. The abiotic average pit depths were 39 and 46 µm, and average pit areas of 1533 and 1939 µm^2^, for the AR and P coupons, respectively. The biotic average pit depths were 47 and 52 µm, with the average pit areas of 1943 and 2398 µm^2^, for the AR and P coupons, respectively. For this study, a pit was classified as having a depth >5 µm and an area >650 µm^2^ ^25^.

**Fig. 3:**
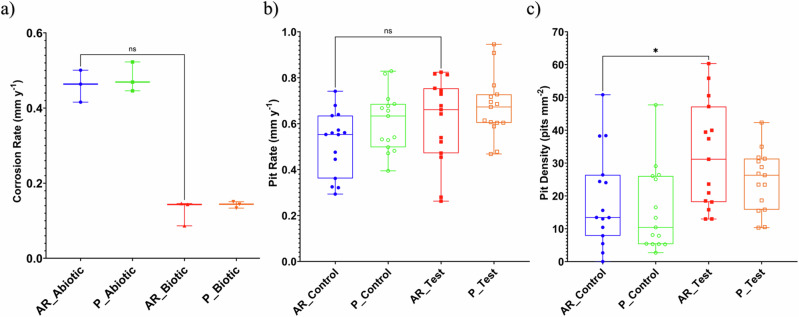
Corrosion metrics for abiotic and biotic conditions. Abiotic and biotic corrosion performance after exposure to anaerobic nutrient-enriched ASW media for 28 days. **a** CR via gravimetric analysis and surface profilometry assessed, **b** pit rate (PR) and **c** pit density (*P* < 0.05), for the AR and P coupons. Corrosion metrics for abiotic and biotic conditions.

Figure 3a provides an evaluation of the carbon steel corrosion rates (with the caveat that such an assessment assumes uniform corrosion). For the abiotic condition, there was a higher corrosion rate when compared to the biotic condition, though there was no significance. Furthermore, when comparing between the two surface roughness types, AR and P coupons within each reactor, there was also no significance (*n* = 3). According to the NACE SP0775-2023 assessment criteria, there was a severe corrosion rate (>0.25 mm yr^–1^) in the abiotic and a moderate corrosion rate (between 0.025 and 0.12 mm yr^–1^) in the biotic (Fig. 3a); whilst a severe pit rate (>0.38 mm yr^–1^) was assessed for both the abiotic and biotic conditions (Fig. 3b)^19^.

Further analysis of the surface profilometries in Fig. 2, allowed a quantitative determination of the pit rate Fig. 3b and pit density Fig. 3c of the carbon steel coupons. For the biotic reactor, though, there was a higher pit rate, although there was no significant difference evident between the abiotic and biotic conditions. Moreover, there was a significant increase in the incidence of pitting. There was an average pit density of 13 and 10 pits mm^–2^ in the abiotic reactor, with 31 and 26 pits mm^–2^ in the biotic reactor, for AR and P coupons, respectively. Again, there were no significant differences when comparing between the two surface roughness types within each reactor.

### Electrochemical measurements

**Fig. 4:**
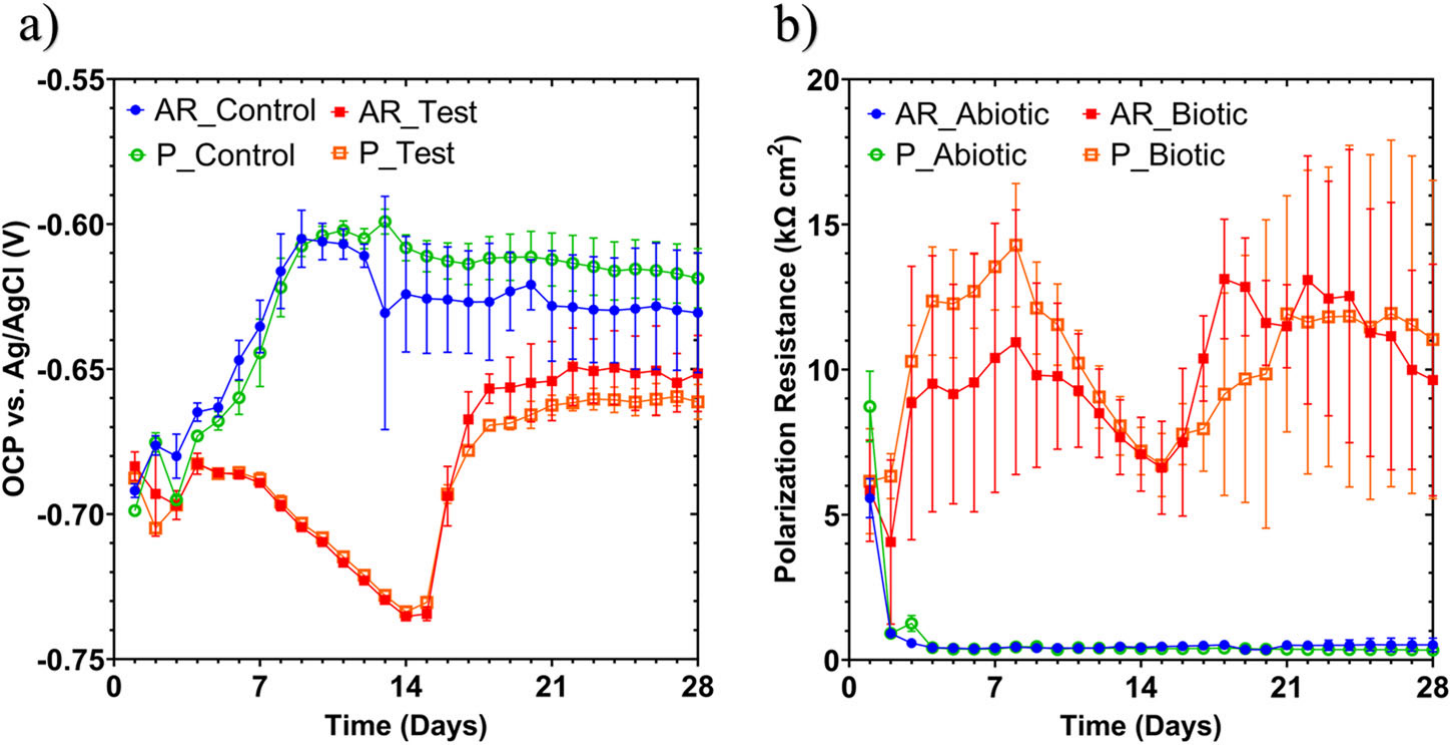
Linear polarization resistance of carbon steel over 28 days for abiotic and biotic conditions. LPR data for UNS G10180 carbon steel: **a** Open-circuit potentials and **b** polarization resistance in anaerobic nutrient-enriched ASW media (abiotic and biotic conditions), for the AR and P coupons (data points represent mean ± standard deviation, *n* = 3). Reactor stirrer at 50 rpm. Linear polarization resistance of carbon steel over 28 days for abiotic and biotic conditions.

Figure 4 shows clear differences between the abiotic and biotic anaerobic nutrient-enriched ASW media for the OCP (i.e., corrosion potential, *E*_corr_, for UNS G10180 carbon steel) and polarization resistance. The abiotic condition, Fig. 4a, had a distinct +0.100 V increase in the *E*_corr_ during the first seven days that can be linked with the presence of a conditioning film (i.e., an adsorbed organic layer) and the formation of an inorganic corrosion product layer. A pseudo-steady state *E*_corr_ was attained in the following 18 days (where AR had a slightly greater variation in potential). Conversely, for the biotic condition, there was a gradual electronegative shift in the *E*_corr_ after seven days, until Day 14, after which *E*_corr_ swiftly increased by +0.070 V. The potentials for both abiotic and biotic in the latter stages (Days 16–28) were generally similar and ranged between –0.610 and –0.660 V vs. Ag/AgCl.

In Fig. 4b the LPR-derived polarization resistance (*R*_p_) after Day 3 remained low at approx. 500 Ω cm^2^ for the sterile abiotic condition_,_ indicative of uniform corrosion across a porous corrosion film. Whereas a periodic response over several days was evident for the biotic condition, with *R*_p_ ranging between 5000 and 15,000 Ω cm^2^. The pioneering bacterial attachment/colonization, biofilm formation and growth kinetics will inevitably lead to a more complex electrochemical response. Overall, there were no significant differences when comparing between the two surface roughness’s (AR and P) within both the abiotic and biotic reactor environments.

**Fig. 5:**
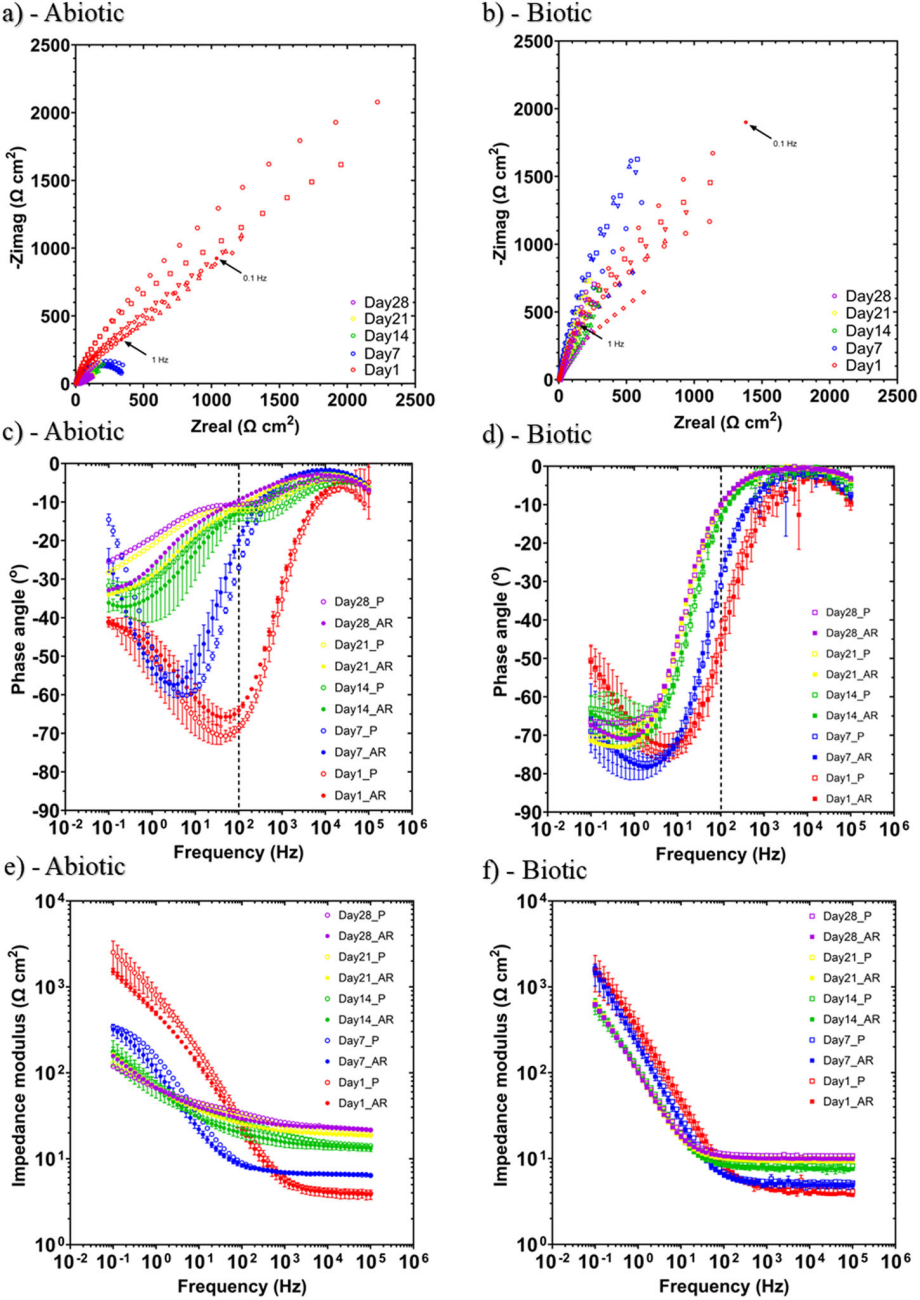
Electrochemical impedance spectroscopy of carbon steel over 28 days for abiotic and biotic conditions. EIS data for UNS G10180 in anaerobic nutrient-enriched ASW media at OCP: **a**, **b** Nyquist, **c**, **d** Bode phase angle (${\theta }$ vs. *f*), and **e**, **f** Bode impedance modulus (|*Z*| vs. *f*) over 28 days. (*n* = 3). Reactor stirrer at 50 rpm. Electrochemical impedance spectroscopy of carbon steel over 28 days for abiotic and biotic conditions.

Figure 5 shows the EIS data for UNS G10180 in the anaerobic nutrient-enriched ASW media presented in three forms: Nyquist, Bode phase angle and Bode impedance modulus plots. The sterile abiotic condition on Day 1 typifies an electrochemical response for the formation of a porous interface, with diffusion of soluble electroactive bacteria across an organic conditioning film^4^ and nascent inorganic corrosion product layer. The diffusive behaviour is associated with linear features having a roughly 45° slope (a Warburg impedance response) and phase angles close to 45° in low-frequency region (10^–2^–10^0^ Hz) (see Fig. 5a, c). At Day 7, a depressed Nyquist semicircle and phase angles tending towards zero are evident, indicative of a more prominent resistive component operating within the low-frequency region. Subsequently, the abiotic impedance spectra shift towards lower frequencies (10^–2^–10^0^ Hz), with a combined diffusive/resistive behaviour. The biotic condition had a consistently uniform EIS response over the 28-day test, with only minor variation in the spectra and suggests the absence of significant detectable electrochemical changes with time. Notably, there are no discernible Nyquist semicircles (Fig. 5b), with Day 1 being more capacitive/diffusive in character related to the well-established double-layer concept (i.e., interfacial charge distribution) and diffusion of electroactive bacteria. Here a wider low frequency region (10^–1^–10^2^) is likely to be subject to a greater influence of adsorption processes, associated with the adhesion of the pioneering bacteria on a conditioning film^26,27^ and biofilm formation.

The EIS spectra were fitted using an equivalent circuit model (ECM) shown in Supplementary Fig. 4. Both the abiotic and biotic data generally had a good fit, with the quantitative fitting results shown in Supplementary Table 4. *R*_s_, *R*_film_ and *R*_ct_ are the solution resistance, the resistance of the biofilm or the corrosion product film, and the charge transfer resistance, respectively. The constant phase element (CPE) characterizes the ‘non-ideal’ capacitance behaviour of either the biofilm or the corrosion product film layer, and the charge transfer capacitance. In Supplementary Table 4, *Q* and *n*, are admittance and exponent parameters from the CPE. For the abiotic reactor, there is a capacitive behaviour over the first week, with a diffusive behaviour over the final three weeks in the film layer (reflecting ion adsorption). Whilst there was a diffusive behaviour in the double layer, which reflects charge transfer, due to the formation of corrosion products (rust, porous oxide layer). The exponent parameter in the double layer reflects a non-ideal capacitance, which is indicative of resistive and inductive parasitics, because of a more prominent resistive component. *R*_film_ is consistently low over the 28 days, and there are no significant changes in *R*_ct_. For the biotic test reactor, there is a capacitive behaviour in both the film layer and double layer due to the presence of the biofilm. There are no significant changes in the *R*_ct_ in the double layer over time. The exponent parameter for the film layer is greater than 0.8 at most time points, which indicates a non-ideal capacitance response. This is true for the first two weeks in the double layer. However, during the final two weeks of the experiment, the exponent parameter is closer to 1 for the AR coupons in the double layer, which indicates a strong capacitive behaviour. The ECM and EIS both have general agreement with the LPR data.

Supplementary Figure 5 shows the potentiodynamic polarization curves for UNS G10180 for the abiotic and biotic reactors in anaerobic nutrient-enriched ASW media after 28 days. Supplementary Table 5 shows the corrosion parameters obtained from the polarization curves. From the Tafel slopes, there is a similar cathodic behaviour (reduction) when comparing the abiotic and biotic ASW media, which is linked to the predominant hydrogen evolution reaction under anaerobic conditions. Conversely, the anodic Tafel slopes (oxidation) are greater, demonstrating almost limiting current densities, in the biotic compared to the abiotic media. Thus, after 28 days the biofilm hindered the iron dissolution reactions. Overall, the abiotic condition had a higher *j*_corr_ compared to the biotic condition. This is consistent with the more uniform corrosion morphology seen for the abiotic coupon surfaces (see Fig. 2). Similarly, the sterile abiotic condition had a more electropositive *E*_corr_ when compared to the biotic condition. The polarization results corroborate the LPR and EIS data, with no significant differences when comparing between the two surface roughness types within each reactor.

### Biofilm characterization

CLSM with differentiation of live and dead biofilm cells was performed and can be found in Supplementary Fig. 6. The heterogeneous biofilm distribution over the surface of the carbon steel coupons did not allow measurements of the maximum biofilm thickness. Therefore, the thickness of biofilms was not determined. It was also difficult to identify significant differences in the structure and distribution of live and dead cells in the biofilms across the two surface roughness types, AR and P coupons, within the biotic reactor. In general, both surfaces had similar live and dead cell ratios (ca. 98% live to ca. 2% dead).

**Fig. 6:**
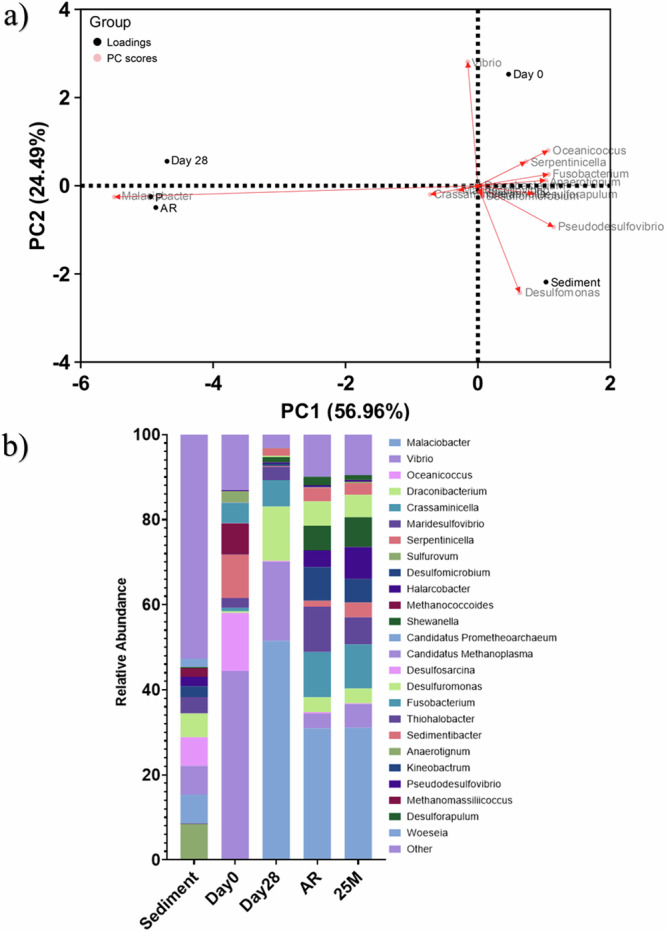
Characterisation of community dynamics using 16S rRNA amplicon sequencing. Principal component analysis biplot (**a**); microbial community. The results show the mean relative abundances of microbial communities classified at the genus level, for the top 25 genera, from 16S rRNA amplicon sequencing (**b**); for environmental marine sediment, Day 0, and Day 28 planktonic samples, AR and P biofilm samples after exposure to anaerobic nutrient-enriched ASW media for 28 days. Characterization of community dynamics using 16S rRNA amplicon sequencing.

Active microorganism evaluation of the environmental marine sediment, the initial and final biotic ASW media planktonic samples (Day 0 and Day 28), and the biotic AR and P biofilms was undertaken via 16S rRNA amplicon sequencing with two target region, V3–4 for bacteria and archaea. A total of 2,422,833 high-quality sequences were obtained after bioinformatics processing of the raw reads. From these, 95.8% was classified for the sediment sample, with 99.99% classified for the Day 0 and Day 28 planktonic samples, AR and P biofilm samples. These sequences were taxonomically classified into microbial genera. The top 25 microbial genera are presented in Supplementary Table 6 in the supplemental material. Figure 6 summarizes the sequencing data, showing a principal component analysis (PCA) (a) and a stacked bar plot (b) illustrating the relative abundances for the top 25 genera. Molecular identification of the microorganisms showed that the initial sediment sample had a very diverse microbial composition. Most genera had low relative abundances <2%. The dominant genera included *Sulfurovum*, *Candidatus Prometheoarchaeum*, *Candidatus Methanoplama*, *Desulfosarcina*, *Desulfuromonas* and *Thiohalobacter*. Interestingly, there were relatively high numbers of archaea in the sediment sample compared to the other samples. The sediment sample had low or negative Spearman correlation coefficients with the other samples which was attributed to changes in conditions such as temperature and media composition from the natural marine environment. There was much less diversity in the Day 0 sample, with *Sulfurovum*, *Candidatus Prometheoarchaeum*, *Candidatus Methanoplama*, and *Thiohalobacter* all exhibiting negligible relative abundances. Whilst genera from *Vibrio*, *Oceanicoccus*, *Serpentinicella* and *Methanococcoides* made up ca. 75% of the relative abundance. Again, the Day 0 planktonic sample had mostly negative Spearman correlation coefficients (Supplementary Fig. 7) with the other samples. After 28 Days, there was a distinct shift in the microbial composition, with substantially lower abundances of methanogenic species. *Malaciobacter*, *Vibrio*, and *Draconibacterium* were the dominant genera making up ca. 80% of the relative abundance. The Day 28 planktonic sample had a Spearman correlation coefficient of 0.11 with the sediment sample, −0.10 with the Day 0 planktonic sample and 0.75 with both biofilm samples. Both biofilm samples exhibited similar microbial populations on both the AR and P coupons. The relative abundances of *Vibrio* decreased to between 3% and 6%, and *Draconibacterium* reduced to ca. 3.5%. *Sulfurovum*, *Candidatus Prometheoarchaeum*, *Candidatus Methanoplama*, and *Thiohalobacter* which were the dominant genera from the sediment sample all had negligible relative abundances in the biofilm samples and Day 28 planktonic sample. Both biofilm samples were similar, with a Spearman correlation coefficient of 0.94. The dominant genera included *Malaciobacter*, *Crassaminicells*, *Maridesulfovibrio*, *Desulfomicrobium* and *Halarcobacter* making up ca. 60% of the relative abundance. There were no methanogenic archaea in the biofilm samples.

The microbial activity was determined by the ATP concentrations (dissolved and total, dATP and tATP, respectively) in both the bulk fluid and biofilms, see Supplementary Fig. 8. It should be noted, that for the sterile abiotic condition, the dATP concentration did not exceed the negative control concentrations, which were used to indicate no ATP activity, and was thus omitted from the figure. For the biotic ASW media (bulk fluid), there was no significant change in the dATP concentration when comparing Day 0 and Day 28, with dATP values on the order of 1 pg mL^−1^. Again, for the sterile abiotic condition, the tATP concentration did not exceed the negative control concentrations and was thus omitted from the figure. Also, for the biotic condition, there was no significant change in the concentration of tATP over the surface of the carbon steel coupons (tATP of about 100 pg mL^−1^). There were no significant differences when comparing between the two surface roughness types within each reactor. As expected, there was a significantly greater ATP concentration for the biotic compared to the abiotic reactor.

## Discussion

Previous studies, which generally investigate single-species biofilms, have shown that bacteria tend to cause an increase in the uniform corrosion rate on carbon steel compared to abiotic controls^8,28–30^. Typical corrosion rates of carbon steel in anoxic environments are reported to be between 0.2 and 0.4 mm yr^−1^ ^14^. As such, carbon steel corrosion is often considered to be insignificant in anoxic environments, such as marine sediments^14,31,32^. However, under either stagnant or low hydrodynamic flow conditions, microorganisms tend to form thicker biofilms on solid surfaces^33^. Ultimately, this results in higher corrosion rates due to microbial corrosion mechanisms, which generate anodic and cathodic regions that affect the passive film on the material surface. Corrosion rates for SRB have been reported to be 0.9 mm yr^−1^ ^34^. During the initial stages of biofilm formation, heterogeneous patches form on material surfaces, inducing the formation of differential concentration gradients. The periodic detachment or formation/growth of the biofilm facilitates the removal of the protective films, which changes the passive film structure and/or increases the dissolution^35^. Additionally, when the biofilm builds up, the diffusion of nutrients from the environment to the microorganisms in the biofilm matrix is limited, which leads to nutrient deprivation. The interfacial microenvironment is altered, changing the diffusivity of metabolites and nutrients through the biofilm layer^34^. Here, the biofilm affects the transportation of chemical species at the steel/ASW interface^35^. This has been shown to cause a shift in metabolism, which exacerbates corrosion rates^29^.

However, studies that have investigated mixed-species biofilms have challenged these observations. Due to the presence of a mixed-species biofilm formed on the material surface, uniform corrosion rates have been shown to decrease^36,37^. The main mechanism of corrosion inhibition is the biofilm-induced formation of green rust (GR) compounds, such as siderite, ${{\rm{GR}}({\rm{CO}}}_{3}^{2-})$, and iron sulfide compounds ${\rm{GR}}({\rm{S}}^{2-})$^36^$.$ The presence of biogenic iron sulfide acts as a barrier by impeding the diffusion of ferrous ions from the metal surface to the aqueous environment^14^. This impediment has been linked to organotrophically grown SRB. Whereas, in organic matter-free cultures, where the predominant corrosion mechanism is EMIC, no significant slowing of corrosion due to the formation of corrosion products in the crust layer has been observed^14,31,32^. In combination with the formation of green rust, the mixed-species biofilm acts as a protective layer which results in decreased uniform corrosion rates^33^. At the same time, the mixed-species biofilm has been shown to aggravate localized pitting corrosion. Here, patchy distribution of the biofilm may be causing differential aeration effects on the steel surface, which in turn affects the oxidation–reduction conditions at the steel/ASW media interface^35^. Through the generation of microenvironments, whether that is primarily influenced by metabolic activity or through physical gradients, localized pitting has been shown to be accelerated^37^.

**Fig. 7:**
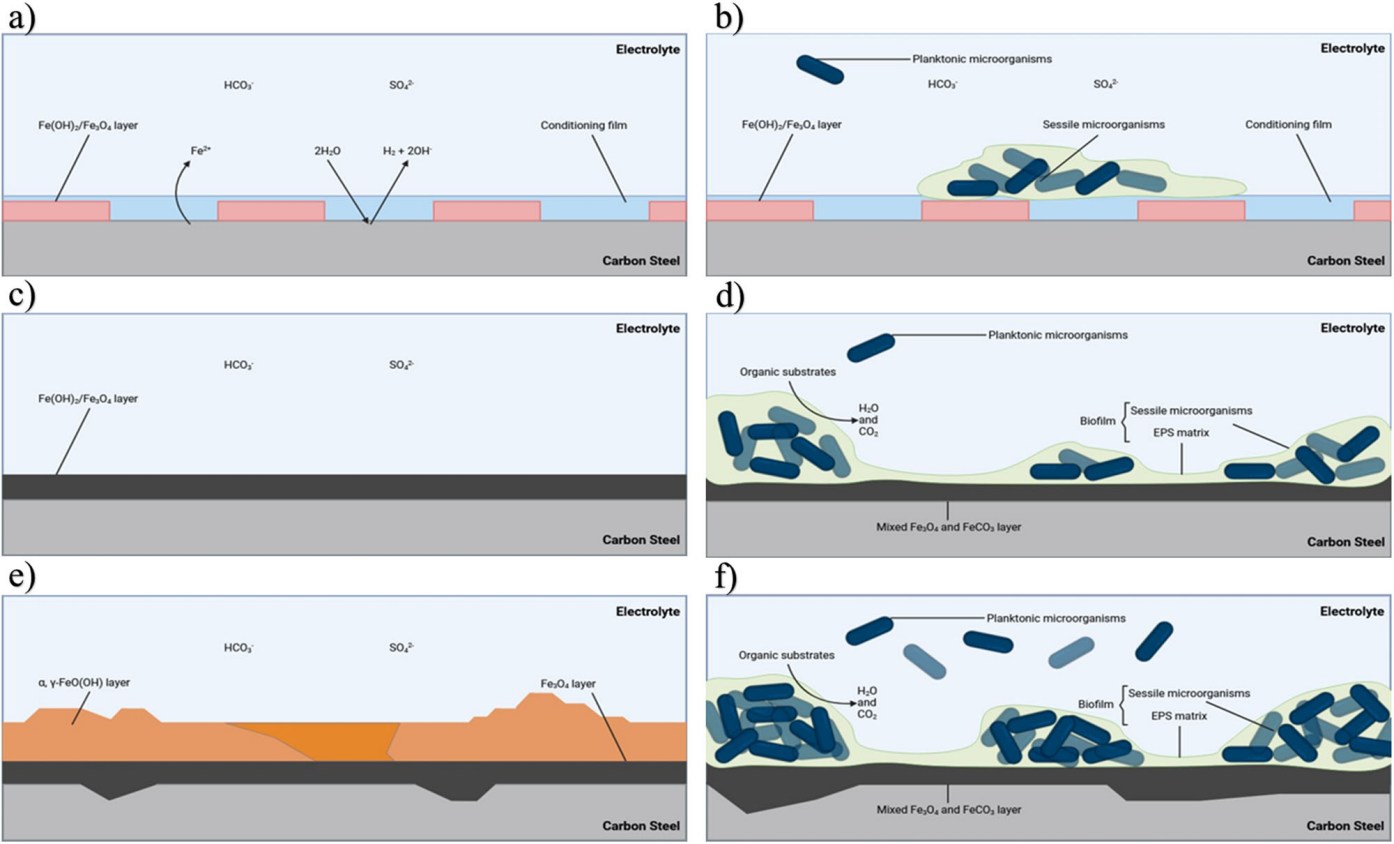
Proposed mechanisms for the initial stages of corrosion for abiotic and biotic conditions. Illustration of the initial stages for UNS G10180 carbon steel in anaerobic abiotic and biotic nutrient-enriched ASW media. Corrosion mechanisms, **a**, **b** the formation of nascent inorganic corrosion film and the organic conditioning film with pioneering bacterial attachment; **c**, **d** maturing corrosion film under the abiotic condition and biofilm growth and colonization under the biotic condition; **e**, **f** uniform and pitting corrosion under patchy corrosion deposits and bacteria clusters. BioRender.com (2023). Proposed mechanisms for the initial stages of corrosion for abiotic and biotic conditions.

Figure 7 provides an illustration for both the abiotic and biotic conditions during the initial stages, as they evolved over time during this present study. For the abiotic control, during the first few days, a conditioning film is developing as redox mediators adsorb onto the surface and a corrosion film is beginning to develop^34,38^. Interestingly, for this study, the different surface roughness types, AR and P, did not appear to impact the rate of corrosion. Rough surfaces have a larger interfacial area with the corrosive environment and therefore an increase in surface roughness was expected to enhance the rate of any corrosion processes. However, the effect of surface roughness is more pronounced under turbulent flow conditions^39^. The low laminar flow conditions used in this study were not conducive to significant differences in corrosion rate.

Under anaerobic conditions, the corrosion of iron produces ferrous hydroxide $({\rm{Fe}}({{\rm{OH}})}_{2})$. This may then transform via the Schikorr reaction into magnetite (${{\rm{Fe}}}_{3}{{\rm{O}}}_{4})$, releasing more hydrogen^40^. This results in anodic polarization over the first week, until the corrosion potential reaches an equilibrium. From equivalent circuit modelling, there is initially a capacitive behaviour observed over the first week, which reflects ion adsorption and the development of a nascent ${\rm{Fe}}({{\rm{OH}})}_{2}/{{\rm{Fe}}}_{3}{{\rm{O}}}_{4}$ film. Subsequently, there was diffusive behaviour during the final three weeks in the film layer. Moreover, there was a diffusive behaviour in the double layer, which reflects charge transfer, due to the formation of corrosion products, such as lepidocrocite $({\rm{\gamma }}-{\rm{FeO}}({\rm{OH}}))$ and magnetite (${{\rm{Fe}}}_{3}{{\rm{O}}}_{4}$). Generally, there was a relatively high level of uniform corrosion of the steel surface for the abiotic media. The presence of ${{\rm{Fe}}}^{3+}$ orange corrosion products suggests that the abiotic condition was not completely anaerobic, and minor oxygen ingress may be an influencing factor here. DO was measured at 2.6 ppm in the abiotic condition and 0.2 ppm in the biotic condition. The presence of the biofilm may have been acting as a passive layer to mitigate uniform corrosion but exacerbate localized pitting. In corrosion systems, a salt film may cover an electrode that is itself covered by a porous oxide layer^41^. This may explain why some localized pitting corrosion was observed in the abiotic media. Additionally, the presence of elemental sulfur, due to the minor oxygen ingress that can oxidize hydrogen sulfide, may have been a contributing factor^42–44^. Sulfate adsorption onto magnetite has been extensively studied^45^ and in the presence of reducing conditions of hydrogen has been shown to be involved in redox reactions to produce iron sulfide and ${{\rm{H}}}_{2}{\rm{S}}$^46^$.$ Cations such as ${{\rm{Ca}}}^{2+}$ have also been shown to enhance sulfate adsorption from alkaline solutions^47^. This may also explain why low sulfide concentrations were observed in the abiotic reactor.

Conversely, in the biotic reactor, an *E*_corr_ shifted electronegative during the first two weeks. This can be attributed to the initial attachment of microorganisms and subsequent biofilm formation on the steel surface^33^. Bacterial cell surfaces also possess net negative electrostatic charge on the outer cell envelope that is exposed to the extracellular environment. Moreover, they are generally negatively charged due to the EPS microenvironment^48^. There was also an initial *R*_p_ increase during the first week that is indicative of biofilm formation on the steel surface, as the presence of biofilm can act as a physical barrier preventing the diffusion of ionic species (e.g., protons) from the environment to the metallic surface^33^. For the biotic reactor, there is a capacitive behaviour in both the film layer and double layer due to the presence of the biofilm. Over the final two weeks, the corrosion potential increased after the biofilm had been established. This is associated with a decrease in the anodic reaction (depolarization) with the growth of a passive film and an increase in the cathodic reaction (polarization) through localized pitting corrosion. Equivalent circuit modelling indicated an ideal capacitive behaviour over the final two weeks. Here, we can infer that there are different electrochemical reactions that are taking place at the interface of the electrode and electrolyte. This may be caused by diffusion limitations due to the biofilm and changes in the prevailing metabolic activity in the system due to the availability of terminal electron acceptors. In general, the biofilm appears to accelerate localized pitting corrosion.

Surface profilometry analysis further highlighted that the biofilm induced greater localized pitting corrosion, with significantly greater pit density, compared to the abiotic control. Generally, the shape and pit morphology were different in coupons exposed to biotic conditions compared to abiotic conditions. Whilst the pit depths were relatively similar in this present study, the average pit sizes and the percentage area of pitting were significantly greater for the biotic reactor. Previous research^8,33,49^ has found that pits were narrow and deep under abiotic conditions, but wide and shallow in the presence of microorganisms^50,51^. In those experiments, it was hypothesized that under biotic conditions, the binding of metal ions and sulfide can act as a protective film on the surface of materials along with the biofilm to prevent proton and chloride attack. Whilst for the abiotic control, there was no such protective film to prevent corrosion of the material^33^. Additionally, previous studies have also suggested that sulfate-reducing bacteria and possibly also the iron-reducing bacteria in anaerobic biofilms participate in the corrosion and rust mineralization of steel in seawater environments^36^. We hypothesize that the deterioration observed in this present study could have been a combination of the patchy distribution of the biofilm formed under flow conditions and the formation of different corrosion products, which will have affected the prevailing oxidation–reduction reactions taking place over the 28 days.

Analysis of the community dynamics revealed a marked change in the predominant relative abundances of microorganisms from the initial sediment sample to the planktonic samples taken from the bulk fluid, and again to the biofilm samples collected from the surface of the coupons taken from the biotic reactor. The dominant genera from the sediment sample were generally anaerobic, halophilic, and obligately chemolithoautotrophic, obtaining energy by oxidizing inorganic compounds (${{\rm{SO}}}_{4}^{2-},\,{{\rm{NO}}}_{3}^{-}$, etc.) through respiration. However, the dominant genera from the planktonic samples taken from the bulk fluid were generally facultative anaerobes known to be chemoheterotrophic, utilizing organic compounds through fermentation. The ASW supplemented with yeast extract clearly had a large impact on the community dynamics. For example, *Methanococcoides*, which had a greater relative abundance in the planktonic samples, is known to be dependent on methylated compounds for nutrition, such as group B vitamins^52^. Another microorganism of interest from the planktonic samples was *Fusobacterium* which has been shown to help facilitate the aggregation and establishment of several other species^53^. This may have played an important role in early biofilm formation^54^. From the biofilm samples, the dominant genera were generally anaerobic, halophilic, sulfate reducing and iron reducing bacteria with a relative increase in the prevalence of electroactive bacteria. Interestingly, there were distinctly lower relative abundances of methanogenic species in both the day 28 bulk fluid and biofilm samples. This can be attributed to inhibition by sulfate-reducing bacteria, as methanogens and sulfate reducers are known to compete for energy sources, with sulfate-reducing microorganisms outcompeting methanogens in sulfate-rich environments^55^. *Malaciobacter*, *Crassaminicells*, *Maridesulfovibrio*, and *Halarcobacter* made up ca. 50% of the relative abundance. These genera are integral to the biogeochemical cycles in marine ecosystems, particularly in sulfur and carbon cycling. Not much is known about the specific functions of *Malaciobacter*. However, like other Proteobacteria in marine environments, they are likely involved in sulfur and nitrogen cycles. Together, these bacteria contribute to maintaining the balance of nutrients and energy flow in marine environments. As previously mentioned, there was an increase in the relative abundance of electroactive bacteria, such as *Desulfomicrobium*, *Shewanella*, and *Desulfuromonas* in the biofilm samples. These electroactive bacteria have been reported to play a key role in EET^9^, which is an important process in MIC. In MIC, sessile cells in a biofilm use metal as an electron donor and a non-oxygen oxidant such as sulfate as the terminal electron acceptor^5^. However, careful consideration needs to be given to both abiotic and biotic corrosion mechanisms that may be taking place.

**Fig. 8:**
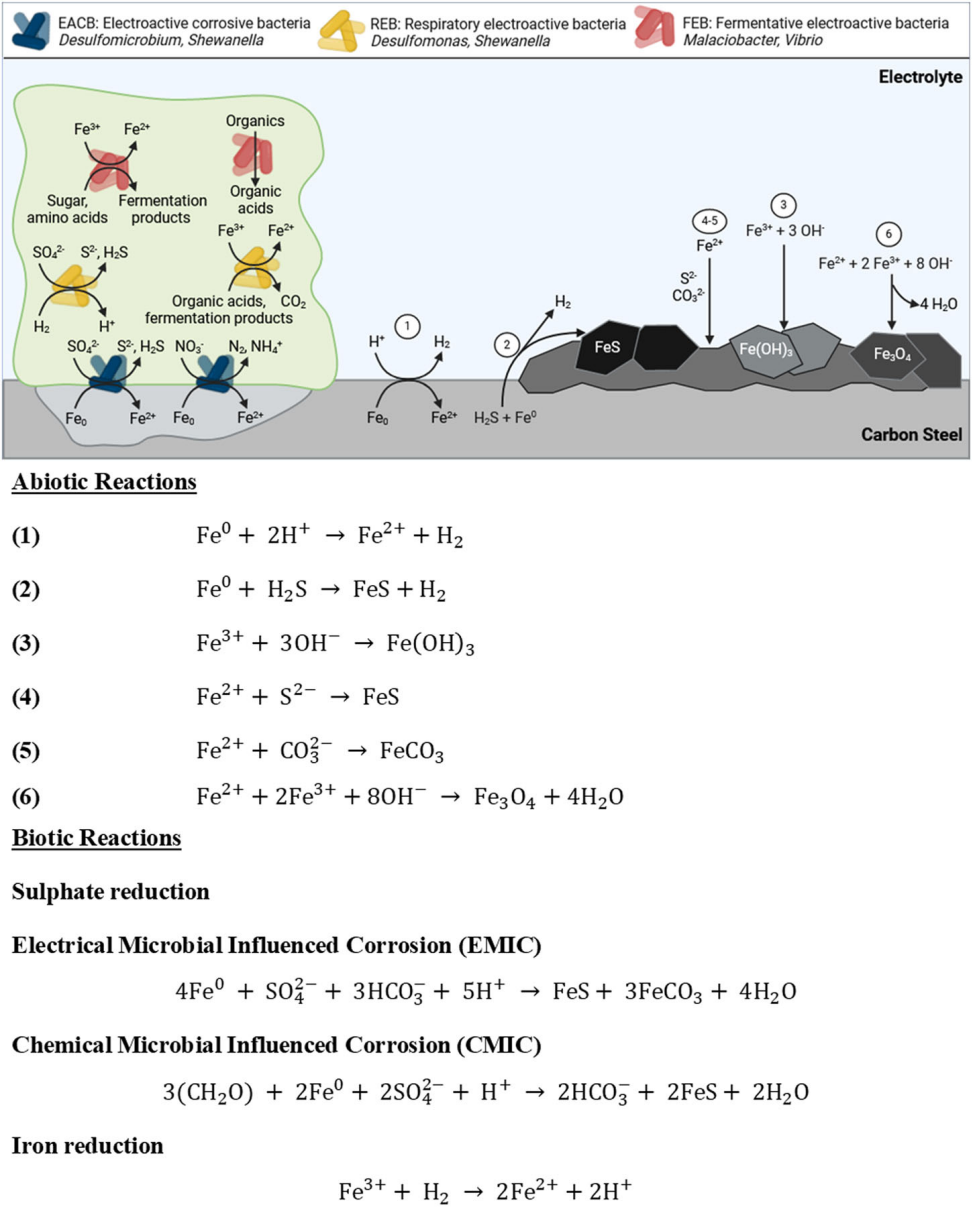
Proposed reaction mechanisms for abiotic and biotic conditions. Overview of key reactions and diversity of microorganisms within a biofilm involved in metal corrosion. It is important to note that the reactions illustrated may occur simultaneously. Numbers 1–6 refer to abiotic reactions associated with corrosion. Biotic reactions involved in the corrosion of ferrous metals are also shown for electroactive corrosive bacteria, electroactive bacteria, and fermentative electroactive bacteria. BioRender.com (2023). Proposed reaction mechanisms for abiotic and biotic conditions.

Under anoxic conditions, both abiotic and biotic corrosion usually leads to the dissolution of ferrous cations (${{\rm{Fe}}}^{2+}$), which precipitate as ${{\rm{Fe}}({\rm{OH}})}_{2}$ and has a reddish-brown appearance. Nonetheless, ${{\rm{Fe}}({\rm{OH}})}_{2}$ is reported to only be metastable in solution and will oxidize to form magnetite (${{\rm{Fe}}}_{3}{{\rm{O}}}_{4}$)^56^ via the Schikorr reaction^40^. The abiotic coupons were observed to have a reddish-brown corrosion product over the 28 days, which was believed to be lepidocrocite. Lepidocrocite has been identified to form in the outer layer on carbon steel coupons permanently immersed in natural seawater^57^. It is difficult to definitively state one abiotic corrosion mechanism as several mechanisms may be occurring simultaneously. Figure 8 highlights several possible abiotic reaction mechanisms^12^. However, the scenario described above for abiotic corrosion changes in the presence of microorganisms, some of which may dramatically accelerate the corrosion kinetics. Figure 8 also illustrates the diversity and heterogeneity of microorganisms within a biofilm that is involved in MIC. We hypothesize possible biotic reaction mechanisms that may be occurring here^9,11,12,14^. Though, it is important to note that through the generation of concentration gradients along with the diffusion of organics, the availability of electron acceptors from the environment and electron donors from the metallic iron, the community dynamics within the biofilm will be constantly shifting. This results in heterogeneity within the biofilm which will ultimately affect the predominant metabolic mechanisms and prevailing corrosion mechanisms^12^.

Over the first two weeks, there appears to have been a shift towards sulfate reduction based on the sulfide microsensor data. After the first week, the reactor had visible black particulates settling at the bottom of the reactor, with the bulk of the fluid being green in appearance. After 2 weeks, there was a visible scum layer, and the reactor was black in appearance with increased turbidity. Biogenic ${{\rm{H}}}_{2}{\rm{S}}$ production will have provided a source of dissolved iron through ${{\rm{Fe}}}^{0}$ oxidation coupled with an increase in the alkalinity of the reactor through consuming protons with the reduction of protons to hydrogen $({{\rm{H}}}_{2})$. For SRB, electrons can either be directly withdrawn from the steel surface through membrane-bound redox proteins via direct electron transfer (DET) or indirectly transferred through redox mediators via mediated electron transfer (MET). We hypothesize that electrical microbial-influenced corrosion (EMIC) was the predominant biotic corrosion mechanism during the first two weeks^11^. Initially, biogenic ${{\rm{H}}}_{2}{\rm{S}}$ can be oxidized by ferric ions (${{\rm{Fe}}}^{3+}$) and thus not be able to precipitate all dissolved iron in the system. However, over the course of the 28 days, the biotic reactor appeared to be black, green in appearance, which was believed to be green rust compounds. This has been identified to form in the inner layer on carbon steel coupons permanently immersed in natural seawater according to references, which is linked to cathodic zones (accelerating cathodic reduction), SRB activity and localized corrosion^57^, such as siderite (${\rm{GR}}({\rm{CO}}_{3}^{2-})$) and iron sulfide compounds (${{\rm {GR}}}({\rm {S}}^{2-})$)^36^. The local microbial sulfate reduction generates alkalinity which helps in increasing the ion activity product (specifically the concentration of carbonate ions) for siderite formation^58^. There was subsequently a steady decline in the concentration of sulfide over the final two weeks of the experiment. When ferric ions (${{\rm{Fe}}}^{3+}$) are available, sulfate reducers such as *Desulfomicrobium* and *Desulfuromonas* spp. may divert electron flux to ${{\rm{Fe}}}^{3+}$ reduction rather than reducing sulfate^9^. Moreover, *Shewanella* sp., which were prevalent in the biofilm samples, have been shown to utilize ${{\rm{Fe}}}^{3+}$ as efficient electron acceptors and are capable of out-competing electron acceptors of lower potential, such as sulfate^9^. Amorphous iron (${{\rm{Fe}}}^{3+}$) oxide compounds can provide a source of ferric ions $({{\rm{Fe}}}^{3+})$. Moreover, iron (${{\rm{Fe}}}^{3+}$) oxides can protect metallic iron $({{\rm{Fe}}}^{0})$ from corrosion because they are an insulating layer preventing DET. Additionally, iron (${{\rm{Fe}}}^{3+}$) oxide compounds can restrict access to organic acids and other sources of protons (${{\rm{H}}}^{+}$), thus limiting ${{\rm{Fe}}}^{0}$ oxidation coupled to ${{\rm{H}}}^{+}$ reduction^12^. However, the formation of corrosion products can also increase the corrosion rate by generating a localized corrosion cell on the surface of the coupon which can exacerbate pitting^11^. When ${{\rm{Fe}}}^{3+}$ reducers remove iron (${{\rm{Fe}}}^{3+}$) oxides, ${{\rm{Fe}}}^{0}$ oxidation coupled to ${{\rm{H}}}^{+}$ reduction is possible again. This allows ${{\rm{H}}}_{2}$ oxidizers, such as sulfate reducers, to resupply protons for iron oxidation^12^. Active microbial iron reduction increases the dissolved iron in the system, which also increases the ionic activity product (specifically the concentration of dissolved iron) favouring siderite precipitation, which we observed by analysing the corrosion products of the test coupons using X-ray diffraction (XRD) (Supplementary Fig. 9)^58^. At the same time, chemical microbial influenced corrosion (CMIC), which refers to the sulphidogenic degradation of organic matter in anoxic environments may be occurring. Here, biogenic sulfide may initially stimulate the anodic part of the corrosion reaction by chemisorption and direct reaction with metallic iron. However, once the metallic surface is covered with inorganic corrosion products such as siderite cathodic reactions become more important drivers of metal oxidation^14^.

The 28 day dual bioreactor protocol represents a pioneering approach to investigating biofilms, specifically tailored to mimic the MIC environment, thus providing a reproducible model for advancing understanding, enhancing predictive measures while employing a multi-disciplinary approach with multiple lines of evidence to comprehensively unravel the complexities of biofilm formation and MIC initiation mechanism. In this study, the dual bioreactor explored the development of MIC on UNS G10180 carbon steel in anaerobic ASW using a marine sediment inoculum (microbial consortia).

- Concurrent electrochemical methods were used to determine when initial biofilm attachment and formation occurred, and the influence that the biofilm had on the surface of the carbon steel coupons. However, whilst these methods provide insights into the redox processes and can quantify corrosion and the influence of the biofilm, they provide limited information about the extent of pitting.
- At the biofilm/steel interface, knowledge of the pitting incidence is critical in the context of a deeper understanding of the MIC initiation and growth processes. Surface profilometry demonstrated the microbial impact when comparing the abiotic and biotic conditions. Coupon analysis showed the biotic media had a significantly greater pit density (*P* < 0.05), with a greater pit depth and size when compared to the sterile abiotic ASW media.
- Furthermore, biofilm characterization using sequencing showed a relative increase in electroactive bacteria, specifically sulfate-reducing and iron-reducing bacteria, such as *Desulfomicrobium*, *Shewanella* and *Desulfuromonas*, within the biofilm when compared to the planktonic species in the bulk fluid. Importantly, these electroactive bacteria play a key role in EET, which is an important MIC process.

From this, we can start to rationalize an idealized representation of the mixed-species biofilm and the microbial mechanisms that lead to the corrosion of carbon steel under anoxic conditions at the electrode/electrolyte interface. Using multiple lines of evidence to gain a holistic understanding of biofilms and MIC, more sustainable prevention and mitigation strategies can be designed. The innovative dual bioreactor protocol allows new insights, establishing a standard model and biofilm-relevant test method for biocide efficacy testing. The development of such a model will support a move towards evidence-based biocide dosing, empowering asset owners to reduce the effect of MIC leading to reduced cost, increased sustainability, and increased asset lifetime.

## Methods

### Test conditions

Two anaerobic Centers for Disease Control (CDC) biofilm reactors (Biosurface Technologies Corporation) were used: an abiotic control reactor and biotic test reactor (key dimensions: 22 cm reactor height and 12 cm internal diameter; 21 cm coupon holder rod; 1.27 cm coupon diameter). Sterile carbon steel coupons were fixed in reactors and exposed to two separate conditions for 28 days. Anaerobic conditions were maintained throughout the test by initially sparging the system with nitrogen gas (oxygen-free nitrogen) (BOC Nitrogen (Oxygen Free), 44-W) over an initial three-day batch phase. ASW (Artificial Seawater) (SwellUK, Aquarium Systems Sea Salt Instant Ocean) supplemented with yeast extract (ThermoFisher, Oxoid, Yeast Extract LP0021) and resazurin solution (0.1%, 0.5 mL L^−1^) (Merck) was used as the growth medium. The test media had the following composition: 18.7 mg L^–1^ Cl^–^, 10.5 mg L^–1^ Na^+^, 2631 mg L^–1^ SO_4_^2–^, 1256 mg L^–1^ Mg^2+^, 400 mg L^–1^ Ca^2+^, 401 mg L^–1^ K, 194 mg L^–1^ HCO_3_^–^, 6 mg L^–1^ B^3+^ 7.5 mg L^–1^ Sr^2+^, and 1000 mg L^–1^ yeast extract (Supplementary Table 1). It was acknowledged that yeast extract contains redox mediators that may adsorb onto the electrode surfaces and chelate metal ions and the test matrix was designed to highlight any significant interference^59^. Resazurin was added as a redox indicator, as it is colourless under oxygen-free conditions but changes to a pink colour in an oxygen-containing environment. Agitation of the reactor baffles was set to 50 rpm to maintain a homogeneous solution. The reactor temperature was at ambient conditions (20 °C). Prior to inoculating the biotic reactor, a 3-day pre-culture was prepared in a blue-cap flask (50 mL) consisting of 10% marine sediment with the remainder of fresh ASW media. The biotic reactor was inoculated using a sterile syringe, where 10% of the working reactor volume (35 mL) was added as the inoculum. Initial adenosine triphosphate (ATP) measurements were taken from the pre-culture and long-term frozen stocks were prepared using 20% glycerol. Supplementary Fig. 4a shows a schematic of the full experimental setup, with Supplementary Fig. 1b illustrating the three-electrode cell setup within each anaerobic CDC biofilm reactor. Both reactors were operated in batch mode for the first three days to allow settlement and facilitate biofilm formation in the biotic reactor. After this period, the reactors were switched to continuous flow of fresh media at a rate of 0.2 mL min^–1^, which replaced about 50% of the 600 mL total volume daily (288 mL day^−1^). The reactors were dosed twice weekly with a biocide on days 4, 7, 10, 13, 16, 19, 22, and 25 using 5 mL of 214–250 ppm of glutaraldehyde (Merck, 340855-1L). The range accounts for the variations in the working volume of the reactors between 300 and 350 mL.

### Microbial consortia

The sheltered zone littoral sediment microbial consortia were collected at a depth between 10 and 15 cm below the sediment surface during low tide from Langstone Harbour, United Kingdom (50°50'11.9“N 0°58'47.5“W). The coastal/estuarine marine sediment (very fine and cohesive mud and silt deposits) was selected to sample microorganisms living under low oxygen conditions. The environmental conditions on the day the marine sediment was collected are presented in Supplementary Table 2. The sediment samples were added to 500 mL of the ASW medium and stored at 37 °C in an anaerobic chamber to maximize the recovery of the diverse microbial populations. Mesophilic bacteria can survive and grow in temperatures between 10 and 50 °C. Thus, a tropic strategy to promote cell growth and viability was employed to maximize microbial recovery. The anaerobic chamber gas mixture consisted of 85% N_2_, 10% CO_2_ and 5% H_2_ (BOC Anaerobic Growth Mix, 290563-L). The composition of the culture medium is described above. Long-term storage of sediment samples and microbial consortia was employed to create frozen stocks at –80 °C.

### Carbon steel coupon preparation

UNS G10180 (AISI 1018) carbon steel disc coupons (Biosurface Technologies—RD128 CS), with dimensions of 12.7 mm diameter × 3.8 mm thickness were used in either the as-received (AR) (*R*_a_ = 1.35652 ± 0.42793) condition or polished (P) (*R*_a_ = 0.44068 ± 0.01887) with a Kemet 15 Lapping machine using 25 μm Type K diamond slurry. The surface profiles and weights for all coupon samples were assessed prior to starting the experiment on Day 0 for surface profilometry and gravimetric analysis to be performed at the completion of the experiment after Day 28. Three-dimensional (3D) surface profiles were taken using a 3D optical profilometer (Alicona imaging infinite focus microscope IFM G4 3.5). A Mettler AT201 was used to take five measurements of the initial weights of all coupons.

### Experimental setup

Before autoclaving, the two anaerobic CDC biofilm reactors were cleaned with detergent and allowed to dry. The empty reactors with attached tubing were placed in autoclavable bags; all-tube openings and air filters (Millex, 0.2 µm) were covered in aluminium foil, with tube openings clamp shut. The empty assembled reactors were autoclaved for 15 mins at 121 °C, along with prepared ASW test media. After cooling the reactors were transferred into a sterilized microbiological safety cabinet, along with all rods, carbon steel test coupons, as well as any sensors and electrodes. Working electrode rods were prepared in advance. For each working electrode rod, wires were soldered to each coupon separately. The coupon face with the soldered wire was then covered with a lacquer solution (Polishing Shop, Type 45 Stop Off Lacquer) and allowed to dry. To assemble the reactors, all rods with coupons were submerged in 99% ethanol for at least 10 s and then inserted into the autoclaved reactors. Any sensors or electrodes used in place of a rod were also inserted, after also being sterilized with 99% ethanol for at least 10 s. The medium bottles and all tubing were connected in a microbiological safety cabinet. Once both reactors were fully assembled, they were transferred to the working area, with access to a N_2_ gas supply. The tubing was evenly split into each reactor to equalize the pressure gradient caused by the peristaltic pump (Matson Marlow 300 series).

### Sulfide analysis

Sulfide concentrations were monitored daily in each reactor using a Unisense, SULF-50 sulfide microsensor (50 μm diameter) and amplifier (Unisense, ${{\rm{H}}}_{2}{\rm{S}}$ UNIAMP). The microsensor measures the partial pressure of ${{\rm{H}}}_{2}{\rm{S}}$ gas, and the total concentration is a function of pH and temperature. The microsensor limit of detection is 0.3 µM, with a range from 0 to 300 µM sulfide in water. Calibration utilized the ${{\rm{H}}}_{2}{\rm{S}}$ and SULF sensor calibration kit (Unisense, CALKIT-${{\rm{H}}}_{2}{\rm{S}}$). Due to the nature of the experimental setup, it was not possible to calibrate the microsensors during the experiment. However, calibrations were performed both prior to starting the experiment and once the experiment had finished to confirm that the sensors were still calibrated. The SensorTrace Suite software was used to collect the sulfide microsensor data. The sensor has a higher signal for zero right after it has been connected to the amplifier, thus each microsensor collected readings for five minutes (~300 data points) on each day. This was to allow the sensor to stabilize.

### Surface profilometry and visual inspection

Corrosion products and biofilms were removed from the surface using the cleaning protocol as described previously for the gravimetric analysis. Three-dimensional (3D) profiling of the carbon steel surfaces was reconstructed using an Alicona imaging infinite focus microscope IFM G4 3.5. The images allowed assessment of changes in surface roughness compared to the surface profiles obtained prior to testing. Additionally, ImageJ/Fiji was used for the quantitative determination of pit depth, width, height, percentage area, and to assess pit rate and pit density. This analysis was performed on fifteen total locations on three coupons (five locations each) for both the AR and P coupons. The method involved applying a colour threshold to depths >5 µm. Then, the images were converted to a binary mask. Next, measurement parameters were selected for areas >650 µm^2^. Finally, the images were analysed to display counts, area, and average size of pits. The pit parameters were adapted from ASTM G48-11^25^. For pit rate analysis, the deepest pits from each image were captured using the Alicona. Pit rates were calculated using the formula described in NACE SP0775-2023^19^.

### Gravimetric analysis

Corrosion products and biofilms were removed following the ASTM G1-03 standard with a 15% inhibited hydrochloric acid described in NACE SP0775-2023^19,60^. A stock solution was made of 37.5% HCl (Merck, Suprapur, 1.00318.0500) to which 10 g/L of 1,3-di-n-butyl-2 thiourea (DBT) (Merck, 8.20423.0250) was added. Immediately prior to use, the stock solution was diluted by slowly adding a measured volume of stock solution to an equal volume of deionised water with stirring. A Mettler AT201 was used to take five measurements of all coupons. Corrosion rates were determined by the gravimetric technique that considers the weight loss and surface area of the metal samples described in NACE SP0775-2023^19^.

### Electrochemical analysis

Electrochemical measurements were performed using a Gamry Instruments potentiostat (Ref 600 Plus). The electrochemical behaviours of the carbon steel coupons were evaluated using a three-electrode system consisting of a UNS G10180 coupon as the working electrode, graphite rod (Alfa Aesar, 99.9995%, 6.15 mm diameter, 152 mm long) as the counter electrode, and a silver/silver chloride (Ag/AgCl, 3.5 M KCl) reference electrode (Sentek, (AgCl) Double junction Reference Electrode). On day 1, after the test reactor was inoculated, both reactors were left for at least 1 h prior to performing any electrochemical measurements. Open-circuit potentials (OCP) were recorded for each coupon on day 1 prior to measuring linear polarization resistance (LPR) and electrochemical impedance spectroscopy (EIS). LPR and EIS were measured daily for each sample. LPR measurements were performed from ±10 mV with respect to *E*_OCP_ using a scan rate of 0.167 mV s^−1^. EIS measurements were performed at OCP with an applied 10 mV_rms_ sinusoidal potential signal with a frequency range of 10^−2^–10^5^ Hz. Potentiodynamic polarization measurements were performed at the end of the experiment on day 28 for each coupon from –0.200 to +0.200 V using the scan rate of 0.5 mV s^−1^. Standard procedures were followed when selecting an equivalent circuit best-fit using the Gamry Echem Analyst software: (i) the chi-squared (*χ*^2^) error was suitably minimized (*χ*^2^ ≤ 10^–4^) and (ii) the errors associated with each element were ranged between 0% and 5%.

### Confocal laser scanning microscopy and post-image analysis

The distribution of live and dead cells within biofilms was studied using confocal laser scanning microscopy (CLSM). Coupons were gently rinsed with sterile anaerobic phosphate-buffered saline (PBS), with the following composition: NaCl 8 g, KCl 0.2 g, Na_2_HPO_4_ 1.44 g, KH_2_PO_4_ 0.245 g, deionised water 1 L. and stained using the FilmTracer Live/Dead biofilm viability kit (Invitrogen) according to the manufacturer’s instructions. Before imaging with a Leica SP8 confocal microscope, coupons were rinsed with sterile deionized water to remove the excess dyes and fixed using Mowiol. Mowiol had the following composition: 2.4 g Mowiol, 6 mL deionized water, 12 mL 0.2 M Tris (pH 8.5), 0.01 g sodium azide, and 6 g glycerol. Images were obtained with a ×63 magnification and glycerol immersion. The dyes used stained live cells with a green-fluorescent colour (SYTO 9) and dead cells with a red colour (propidium iodide). The *z*-stacked images were analysed using Imaris software (Oxford Instruments).

### Microbial community analysis

After 28 days, three AR and three P coupons were gently rinsed with PBS and then placed in a Falcon tube containing 10 mL of ASW solution. Long-term frozen stocks were prepared using 20% glycerol for the bulk fluid, AR biofilm, and P biofilm samples from the biotic reactor. The sediment, three-day pre-culture, day 28 bulk fluid, AR biofilm and P biofilm frozen stocks were sent in triplicate for DNA extraction and 16S rRNA amplicon sequencing. Library preparation and sequencing were performed for the V3 and V4 regions of the 16S rRNA gene targeting both bacteria and archaea. The microbiome analysis pipeline, along with DNA extraction, was performed by Eurofins Genomics LLC. Taxonomic classification method using Kraken2 (v 2.1.1). Bioinformatics and data analysis was performed using the Qiime2 (version 2023.5) software. To visualize the multivariate dispersion of the community composition, a principal component analysis (PCA) analysis was conducted employing GraphPad (version 10.0.2).

### ATP assay

The ATP concentration in both the abiotic and biotic reactors was determined by luminescence after reaction with luciferin-luciferase using the BacTiter-Glo™ Microbial Cell Viability Assay kit (Promega). The assay provides a method for determining the number of viable microbial cells in culture based on quantitation of the ATP present. ATP is the energy source of all living cells and is involved in many vital biochemical reactions. When cells die, they stop synthesizing ATP and the existing ATP pool is quickly degraded. Higher ATP concentration indicates a higher number of living cells. All assays were performed according to the manufacturer’s instructions. Six coupons, three AR and three P, were gently rinsed with PBS and then immersed in a Falcon tube containing 10 mL of ASW solution. Any cells were detached from the metal coupons using a cell scraper (Biologix). Both planktonic and sessile samples were processed with the BacTiter-Glo™ Microbial Cell Viability Assay kit, which measures ATP from as few as 10 microbial cells. The ATP concentrations were determined by measuring luminescence with a Clariostar Plus Multimode Microplate Reader (BMG Labtech). Planktonic cells in each reactor were determined following the same method described before; in this case, 10 mL of the bulk test solution was processed with the BacTiter-Glo™ Microbial Cell Viability Assay kit. Negative controls of PBS, deionised water and ASW solution were used to indicate no ATP activity.

### Corrosion product analysis

Analysis of the corrosion products and biofilms were performed by X-ray diffraction spectroscopy (XRD) using a Rigaku SmartLab thin film and materials diffractometer. Four coupons were extracted from each reactor on day 28, two AR and two P, and stored and allowed to dry under anaerobic conditions. Spectra were collected using a Rigaku SmartLab thin film and materials diffractometer.

## Supplementary information

Supplementary information

## Data Availability

The data that support the findings of this study are not openly available due to reasons of sensitivity and are available from the corresponding author upon reasonable request. Data are located in controlled access data storage at the University of Southampton.
