# Supplementary material for: Dual anaerobic reactor model to study biofilm and microbiologically influenced corrosion interactions on carbon steel
Source: Npj Mater Degrad. 2024 Dec 6;8(1):125. doi: 10.1038/s41529-024-00542-x (PMC11621017; doi:10.1038/s41529-024-00542-x)
Supplement: Supplementary file 1 — Supplementary information [file 41529_2024_542_MOESM1_ESM.pdf]

Supplementary Material

Abiotic Coupons

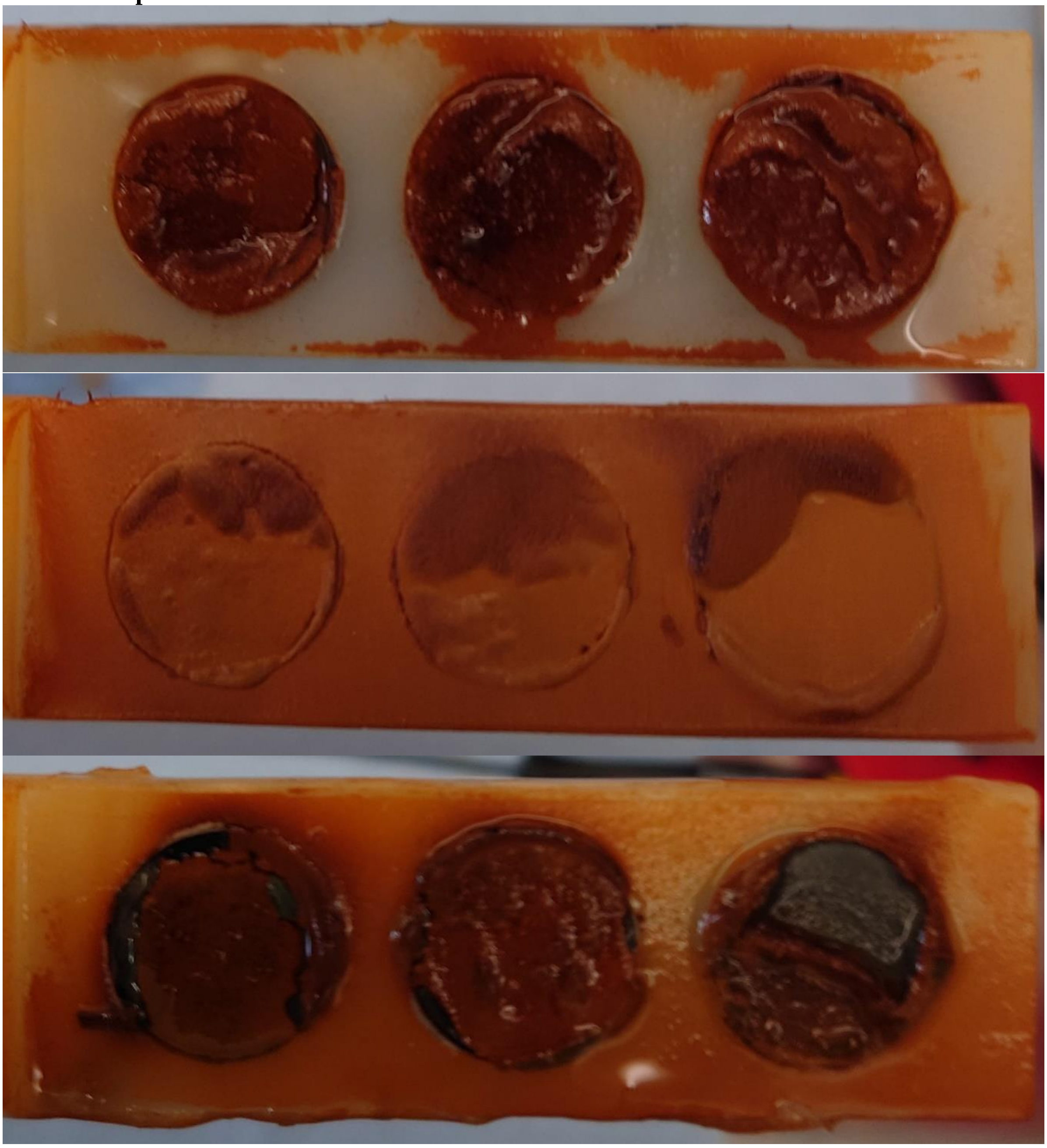

Supplementary Figure 1a. Photographs taken of the coupon rods taken from the abiotic condition on Day 28, on dismantling the reactor, after exposure to anaerobic nutrient-enriched ASW media for 28 days.

Biotic Coupons

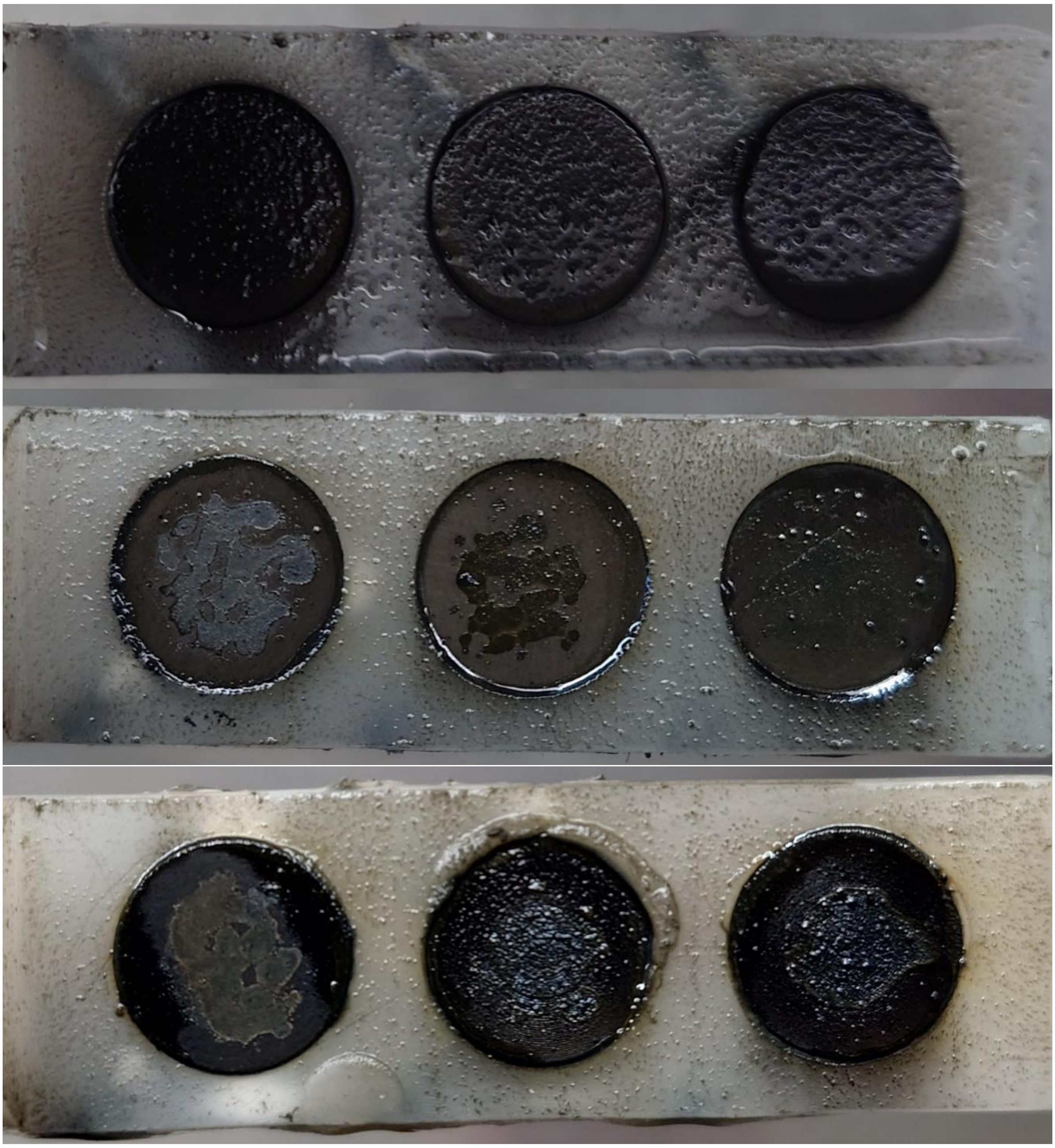

Supplementary Figure 1b. Photographs taken of the coupon rods taken from the biotic condition on Day 28, on dismantling the reactor, after exposure to anaerobic nutrient-enriched ASW media for 28 days.

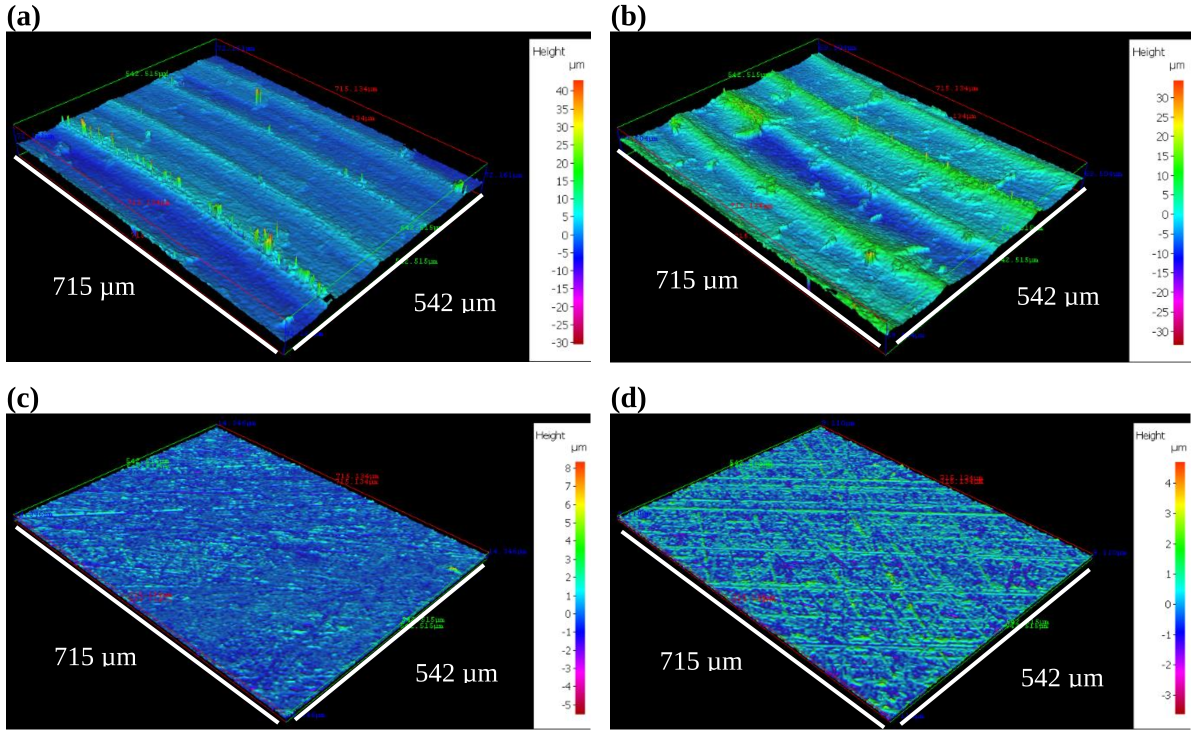

Supplementary Figure 2. Three-dimensional optical surface profilometry of UNS G10180 surfaces at day 0. AR coupons for: **(a)** abiotic and **(b)** biotic conditions; and P coupons for: **(c)** abiotic and **(d)** biotic conditions, prior to exposure to anaerobic nutrient-enriched ASW media for 28 days.

Supplementary Table 1a. Quantitative surface roughness profiles for as-received (AR) coupon samples on day 0 and day 28. R_a average roughness of profile, R_t maximum peak to valley height of roughness, R_z mean peak to valley height, R_p maximum peak height, R_v maximum valley height, R_c mean height of profile irregularities, R_{sm} mean spacing of profile irregularities, R_t/R_z extreme scratch/peak value of roughness profile (higher values (>=1) represent larger scratches/peaks).

Day	Reactor	R_a (µm)	R_t (µm)	R_z (µm)	R_p (µm)	R_v (µm)	R_c (µm)	R_{sm} (µm)	R_t/R_z (µm)
0	Abiotic	1.3±0.7	19.5±11.5	10.3±4.8	12.1±7.3	7.4±7.9	9.1±7.6	193±202	1.8±0.6
	Biotic	1.4±0.8	12.4±6.9	8.3±3.6	8.1±5.2	4.3±2.5	6.5±3.8	156±128	1.5±0.4
28	Abiotic	2.2±0.7	33.6±16.0	17.2±6.8	10.7±5.2	22.9±12.0	18.4±12.5	248±167	1.9±0.5
	Biotic	3.5±1.2	48.3±19.0	26.0±8.7	15.4±5.1	32.9±15.1	27.6±11.7	328±235	1.9±0.4

Supplementary Table 1b. Quantitative surface roughness profiles for polished (P) coupon samples on day 0 and day 28.

Day	Reactor	R_a (µm)	R_t (µm)	R_z (µm)	R_p (µm)	R_v (µm)	R_c (µm)	R_{sm} (µm)	R_t/R_z (µm)
0	Abiotic	0.4±0.03	4.6±0.8	3.5±0.3	2.5±0.5	2.1±0.5	1.9±0.2	38±5	1.3±0.1
	Biotic	0.4±0.03	4.2±0.5	3.4±0.2	2.3±0.3	1.9±0.4	1.9±0.2	36±5	1.3±0.1
28	Abiotic	2.1±0.8	40.6±18.7	18.6±7.0	9.2±3.6	31.4±15.2	24.8±18.8	304±180	2.1±0.5
	Biotic	2.9±0.9	51.8±11.7	26.1±7.0	14.1±3.2	37.8±11.6	34.5±11.1	350±239	2.1±0.4

Supplementary Figure 3. Equivalent circuit model used to generate EIS parameters.

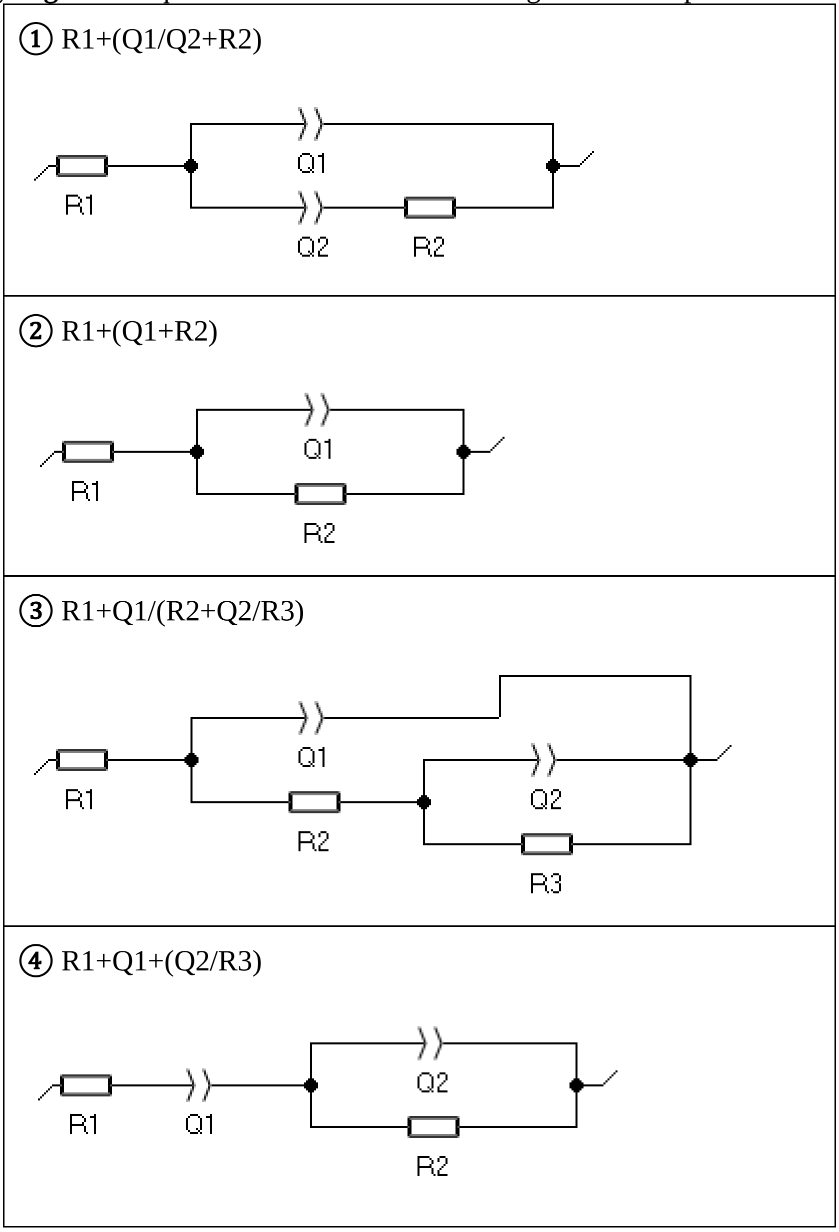

Supplementary Table 2a. EIS parameters of the carbon steel coupons immersed in ASW+YE media for the abiotic control reactors on days 1, 7, 14, 21 and 28. **Equivalent circuit:** ① – R_s+(Q_1/Q_2+R_{ct}); ② – R_s+(Q_1/R_{ct}); ③ – R_s+Q_1/(R_{film}+Q_2/R_{ct}).

Day	Coupon	R_s / Ω cm^2	Q_1 / mΩ^{-1} cm^{-2} s^n	n_1	R_{film} / Ω cm^2	Q_2 / mΩ^{-1} cm^{-2} s^n	n_2	R_{ct} / Ω cm^2	χ^2 ×10^{-4}
1	AR ①	3.9±0.5	0.2±0.03	0.85±0.01	–	0.8±0.9	0.45±0.03	304±52	14
	P ①	4.0±0.2	0.1±0.01	0.87±0.01	–	0.6±0.3	0.45±0.07	612±179	18
7	AR ②	6.7±0.1	2.2±0.1	0.78±0.04	–	–	–	417±24	8.5
	P ②	6.6±0.1	1.2±0.1	0.81±0.01	–	–	–	374±14	3.4
14	AR ①	13.0±1.7	5.3±0.9	0.40±0.03	–	4.0±0.1	0.58±0.03	23±374	2.9
	P ③	13.0±0.6	3.6±2.1	0.42±0.08	28±11	3.4±0.9	0.60±0.04	903±409	2.9
21	AR ③	13.0±8.5	4.3±4.0	0.51±0.21	46±43	4.6±1.3	0.56±0.05	1454±485	3.2
	P ③	18.0±1.5	3.6±1.7	0.42±0.06	39±0.1	8.0±2.1	0.52±0.09	775±287	4.8
28	AR ①	13.6±5.8	3.2±5.6	0.59±0.22	–	4.2±6.7	0.59±0.28	84±124	3.7
	P ③	19.9±1.4	11.0±6.6	0.48±0.10	243±178	9.3±3.7	0.42±0.16	123±162	4.8

Supplementary Table 2b. EIS parameters of the carbon steel coupons immersed in ASW+YE media for the biotic test reactors on days 1, 7, 14, 21 and 28. **Equivalent circuit:** ④– R_s+Q_1+(Q_2/R_{ct}).

Day	Coupon	R_s / Ω cm^2	Q_1 / mΩ^{-1} cm^{-2} s^n	n_1	Q_2 / mΩ^{-1} cm^{-2} s^n	n_2	R_{ct} / Ω cm^2	χ^2 ×10^{-4}
1	AR ④	4.0±0.1	0.6±0.2	0.85±0.02	1.4±0.6	0.77±0.01	2509±1497	41
	P ④	3.7±0.9	0.4±0.3	0.93±0.06	0.9±0.1	0.69±0.20	1342±1251	41
7	AR ④	5.0±0.4	1.7±0.8	0.86±0.02	2.3±0.4	1.02±0.06	616±79	21
	P ④	5.3±0.2	1.5±0.3	0.94±0.07	2.0±1.0	0.92±0.09	939±465	21
14	AR ④	7.7±0.6	3.0±0.6	0.84±0.02	5.4±1.8	0.98±0.04	164±109	13
	P ④	8.2±0.2	3.3±0.6	0.88±0.03	3.7±0.5	0.85±0.02	206±66	15
21	AR ④	9.3±0.7	6.5±6.7	0.80±0.03	7.1±6.1	1.01±0.06	1506±2474	4.8
	P ④	10.0±0.3	2.5±0.4	0.87±0.04	8.1±3.8	0.91±0.09	97±105	5.0
28	AR ④	10.0±0.3	2.9±0.4	0.84±0.01	8.5±4.4	1.02±0.06	156±106	3.5
	P ④	10.8±0.3	3.1±1.2	0.87±0.04	6.6±4.3	0.91±0.13	163±161	4.2

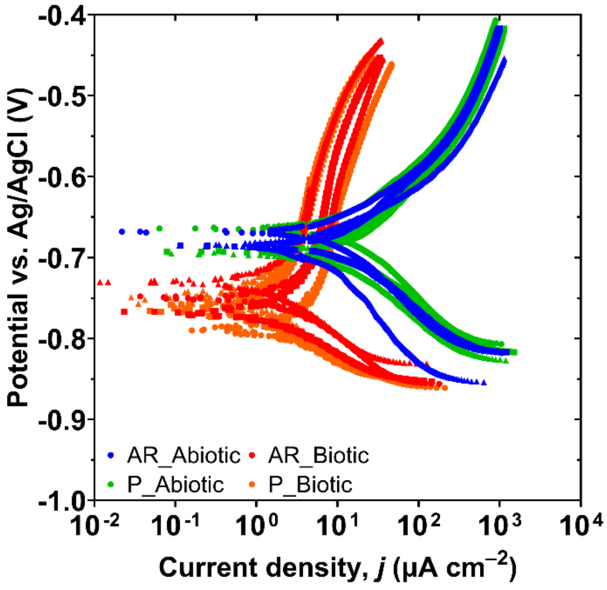

Supplementary Figure 4. Potentiodynamic polarization curves for the abiotic and biotic AR and P UNS G10180 carbon steel coupons at ambient temperature after exposure to anaerobic nutrient-enriched ASW media for 28 days. Scan rate of 0.5 mV s^{-1} and reactor stirrer at 50 rpm. Dissolved oxygen levels were 2.6 ppm (abiotic) and 0.2 (biotic), at Day 28.

Supplementary Table 3. Fitted electrochemical parameters from polarization curves; comparison between the abiotic and biotic AR and P UNS G10180 carbon steel coupons after exposure to anaerobic nutrient-enriched ASW media for 28 days.

	Coupon	j_{corr} / μA cm^{-2}	E_{corr} *vs.* Ag/AgCl / V	β_a (mV dec^{-1})	β_c (mV dec^{-1})
**Abiotic**	AR	14.5 ± 4.8	-0.679 ± 0.010	123 ± 20	146 ± 11
	P	22.1 ± 5.3	-0.684 ± 0.017	129 ± 32	108 ± 144
**Biotic**	AR	2.99 ± 1.04	-0.749 ± 0.019	317 ± 24	86 ± 9
	P	1.93 ± 1.47	-0.769 ± 0.019	263 ± 53	66 ± 6

(a) **(b)**

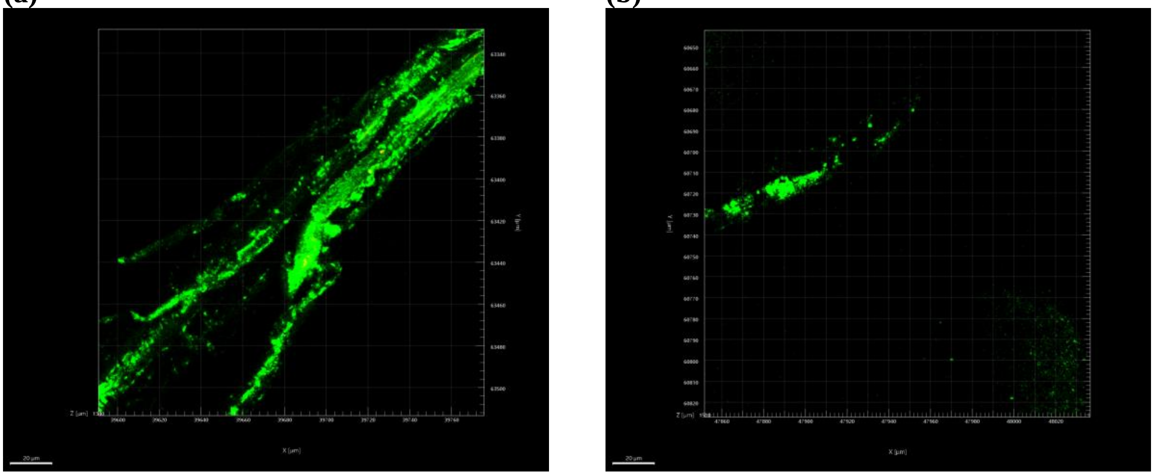

Supplementary Figure 5. Confocal images of biofilms formed over UNS G10180 carbon steel surfaces for **(a)** AR and **(b)** P biofilms, after exposure to anaerobic nutrient-enriched ASW media for 28 days.

Supplementary Table 4. List of top 25 microbial genera identified through 16S rRNA amplicon sequencing with two target region, V3-4 for bacteria and archaea.

Name	Sediment	Day0	Day28	AR	25M
Malaciobacter	0.002629	0.000723	51.51	30.96	31.06
Vibrio	0.1229	44.44	18.57	3.456	5.58
Oceanicoccus	0.02797	13.6	0.2062	0.3402	0.1696
Draconibacterium	0.0827	0.4161	12.77	3.469	3.492
Crassaminicella	0.006454	0.8045	6.202	10.68	10.35
Maridesulfovibrio	0.02844	2.335	3.183	10.64	6.391
Serpentinicella	0.004303	10.18	0.2291	1.389	3.472
Sulfurovum	8.113	0.002169	NA	0.000522	0.000689
Desulfomicrobium	0.06956	0.06236	0.5023	7.878	5.54
Halarcobacter	0.001434	NA	0.2581	3.965	7.52
Methanococcoides	0.08151	7.294	NA	NA	NA
Shewanella	0.01864	0.0103	1.287	5.786	6.979
Candidatus Prometheoarchaeum	6.812	0.015	NA	NA	NA
Candidatus Methanoplasma	6.768	0.03362	NA	NA	NA
Desulfosarcina	6.685	0.003615	NA	0.008087	0.003102
Desulfuromonas	5.615	0.001446	0.3122	5.735	5.274
Fusobacterium	0.001195	4.787	0.001397	0.00287	0.008271
Thiohalobacter	3.865	0.001084	NA	NA	NA
Sedimentibacter	NA	0.06091	1.74	3.18	2.776
Anaerotignum	0.001195	2.691	0.01886	0.1283	0.3395
Kineobactrum	2.52	0.000904	NA	NA	NA
Pseudodesulfovibrio	2.237	0.188	0.03544	0.4844	0.3563
Methanomassiliicoccus	2.058	0.003976	NA	NA	NA
Desulforapulum	0.2462	0.003796	0.000524	1.978	1.161
Woeseia	1.962	0.000362	NA	NA	0.000345

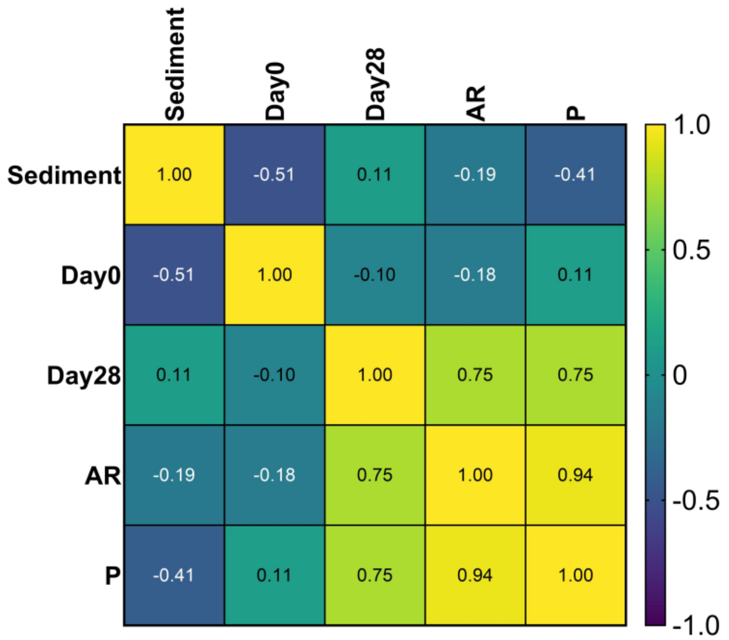

Supplementary Figure 6. Spearman correlation coefficients for environmental marine sediment, Day 0, and Day 28 planktonic samples, AR and P biofilms, after exposure to anaerobic nutrient-enriched ASW media for 28 days.

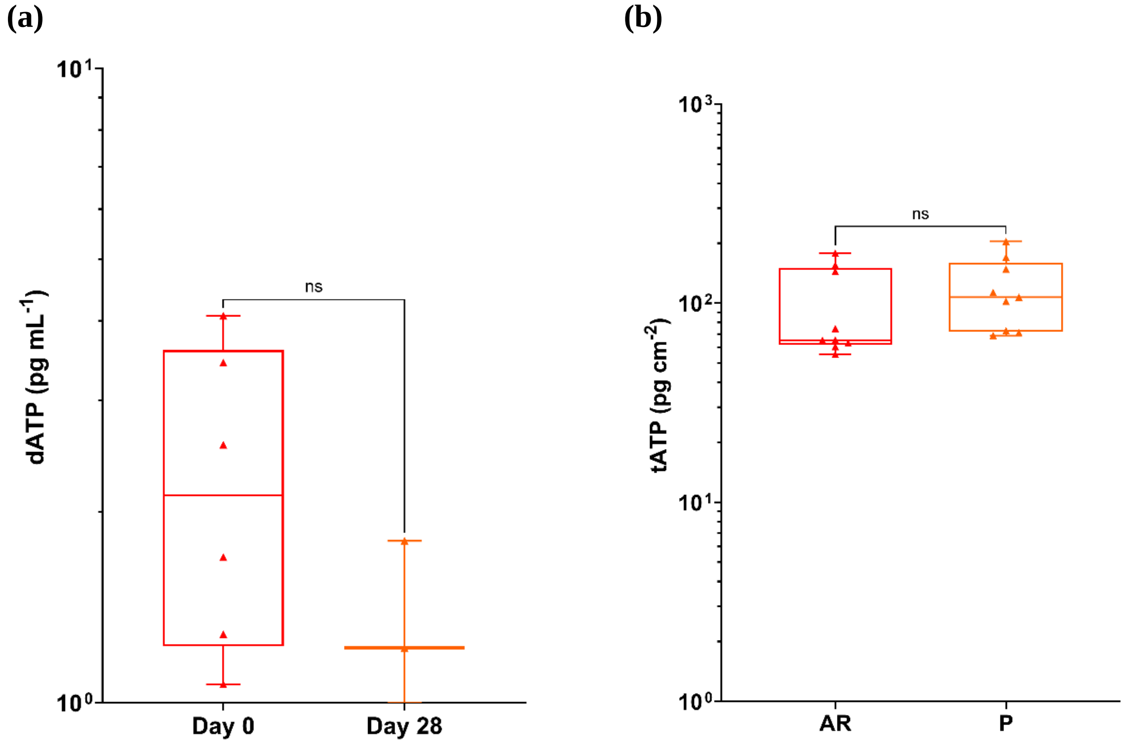

Supplementary Figure 7. (a) Dissolved ATP (dATP) concentrations comparing the anaerobic nutrient-enriched ASW media taken on Day 0 and Day 28 and **(b)** Total ATP (tATP) concentration comparing the biofilm of the AR and P coupons, from the biotic condition, after exposure to anaerobic nutrient-enriched ASW media for 28 days.

(a)

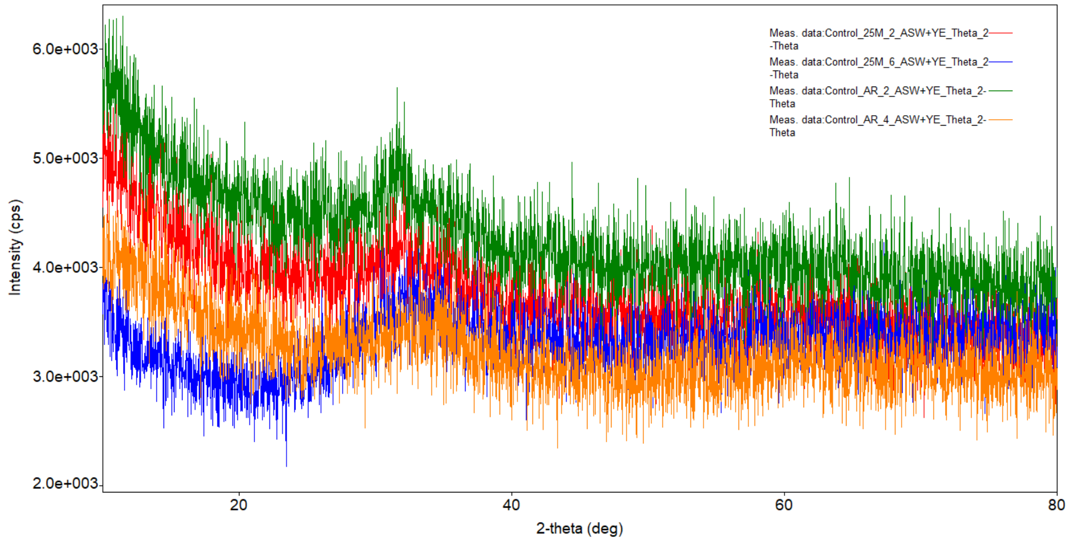

(b)

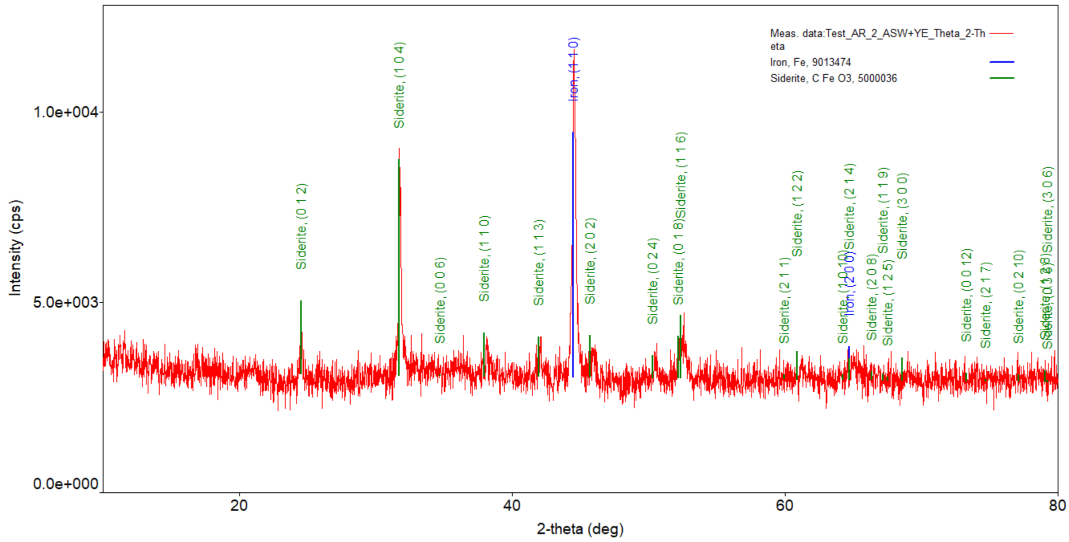

(c)

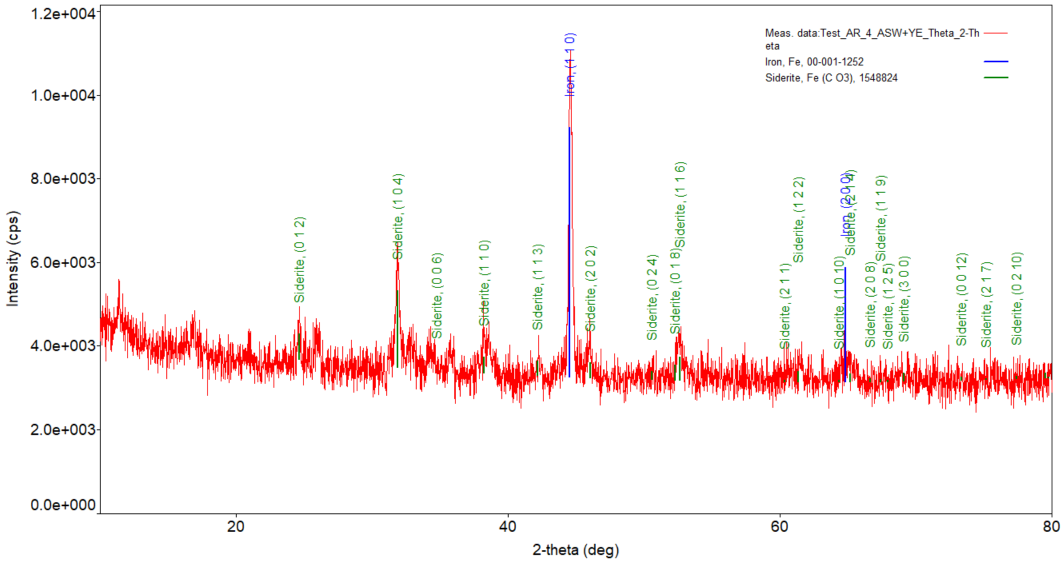

Supplementary Figure 8. XRD patterns for corrosion products formed on carbon steel for abiotic **(a)**, and biotic coupons **(b, c)**, after exposure to anaerobic nutrient-enriched ASW media for 28 days.

Supplementary Table 5. Artificial seawater test solution/media composition supplemented with 1000 mg L^{-1} of yeast extract. Yeast extract has a protein content of about 50%, of which about 20% is glutathione, 6% is nucleic acid. It is rich in 18 kinds of amino acids, functional peptides glutathione, dextran, mannan, trehalose, flavouring nucleotide, B vitamins, biotin, trace elements and volatile aromatic compounds and other components. Calcium, phosphorus, and trace element contents (μg g^{-1}) in yeast extract: Calcium 1120, Phosphorus 18020, Zinc 190, Iron 162, Chromium 5, Potassium 9300, Cobalt 1.2, Manganese 15, Strontium 3.5, Magnesium 2150.

Major Ion	% Total Weight	Concentration (mg L^{-1})
Chloride, Cl-	47.47	18.74
Sodium, Na+	26.28	10.454
Sulfate, SO42-	6.6	2631
Magnesium, Mg2+	3.23	1256
Calcium, Ca2+	1.013	400
Potassium, K	1.015	401
Bicarbonate, HCO3-	0.491	194
Boron, B3+	0.015	6
Strontium, Sr2+	0.001	7.5
Solids Total	86.11%	34.09
Water	13.88	
Total	99.99%	

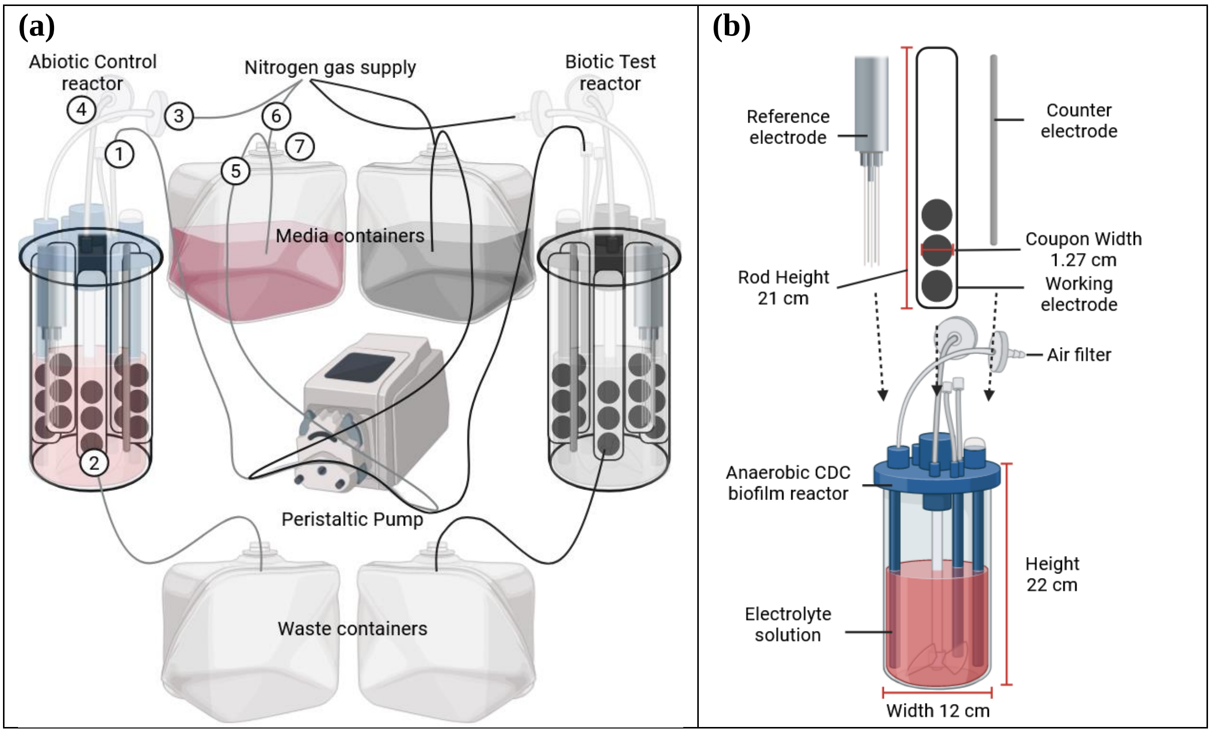

Supplementary Figure 9. (a) The dual anaerobic biofilm reactor system (abiotic and biotic reactors) comprising 10 L media containers, peristaltic pump, magnetic stirrer/hot plate, sulphide microsensor, and the three electrode cell setup. Each reactor has five rods, with three coupons in each rod (15 coupons in total). Each reactor has four inlets. ① The first inlet is connected to the peristaltic pump and then the media container. ② The outlet is connected to the waste containers. ③ Connection to the nitrogen gas source. ④ Air filter (Millex, 0.2 µm) that acts as the exit for excess gas in the reactors. ⑤ The 10 L media container is connected via the peristaltic pump and feeds the first inlet in the reactor. ⑥ Connection to the nitrogen gas source. ⑦ Air filter (Millex, 0.2 µm) that acts as the exit for excess gas in the media containers. **(b)** detailed three-electrode cell setup in an anaerobic CDC biofilm reactor: There are three separate carbon steel coupon working electrodes that can be measured per rod. Each reactor had two rods that were modified for electrochemical analysis, *n* = 3 for both as-received (AR) and polished (P) UNSG10180 carbon steel coupons. Created by BioRender.com.

Supplementary Table 6. Environmental conditions on the day the marine sediment was collected on September 14, 2022.

Date & Time	14/09/2021 07:32	14/09/2021 07:47	14/09/2021 08:02	14/09/2021 08:17	14/09/2021 08:32	14/09/2021 08:47	14/09/2021 09:02	14/09/2021 09:17	14/09/2021 09:32
pH units (pH/ORP)	7.99	7.99	7.99	7.98	7.98	8	8.01	8	8.01
Specific Conductivity mS/cm (Conductivity)	40.982	39.918	39.798	40.598	40.859	39.9	39.561	39.803	38.604
Turbidity NTU (Turbidity/Brush)	265.1	233	663	170.8	205.9	244.6	317.4	610	586
Barometric Pressure mmHg (Necessary Input)	766.7	766.7	766.7	766.7	766.7	766.7	766.7	766.7	766.7
DO mg/L (Hach LDO)	7.41	7.34	7.43	7.25	7.37	7.41	7.45	7.4	7.43
Depth meters (Depth 25 meter)	0.197	0.203	0.199	0.194	0.196	0.194	0.192	0.202	0.194
Turbidity mV (Turbidity/Brush)	256.553	228.405	605.772	173.86	204.651	238.627	302.424	558.721	538.339
DO %SAT (Hach LDO)	91.7	90.5	91.5	89.6	91.3	91.5	91.9	91.4	91.2
ORPAgCl mV (pH/ORP)	220	222	220	216	219	216	220	220	218
Temperature °C (Temperature)	18.5	18.53	18.51	18.55	18.57	18.61	18.63	18.62	18.55
Total Dissolved Solids g/L (Conductivity)	26.228	25.548	25.471	25.983	26.149	25.536	25.319	25.474	24.706
Specific Gravity (Depth 25 meter)	1.019	1.018	1.018	1.018	1.018	1.018	1.018	1.018	1.017
Density kg/m3 (Depth 25 meter)	1018.52	1017.94	1017.88	1018.3	1018.44	1017.91	1017.72	1017.85	1017.23
Salinity psu (Conductivity)	26.32	25.56	25.48	26.05	26.23	25.55	25.31	25.48	24.63
Chlorophyll a µg/L (Chlorophyll a)	5.99	6.2	2.41	2.26	9.23	2.25	2.4	2.47	2.14
